# Representative Residential Property Model—Soft Computing Solution

**DOI:** 10.3390/ijerph192215114

**Published:** 2022-11-16

**Authors:** Aneta Chmielewska, Małgorzata Renigier-Biłozor, Artur Janowski

**Affiliations:** 1 Institute of Spatial Management and Geography, Faculty of Geoengineering, University of Warmia and Mazury in Olsztyn, 10-719 Olsztyn, Poland; 2 Institute of Geodesy and Civil Engineering, Faculty of Geoengineering, University of Warmia and Mazury in Olsztyn, 10-719 Olsztyn, Poland

**Keywords:** representative property model, residential environment, RST, SOMs, soft computing methods

## Abstract

Residential properties are a major component of the environment and economy and a key element for the quality of human life. Faced with disruptive ideological and technological changes in the world, real estate analysis has also become a key research problem for many academic centers and private institutions. Due to the complex nature of properties, they are one of the most difficult and troublesome subjects of analysis. Given the rapid advancements in competitive automated analytical models, the problem of data representative sample selection may prove to be a very wide-reaching subject. The aim of this paper was the assessment of the particular soft computing methods’ (e.g., Self-Organizing Maps, Rough Set Theory) usefulness for selecting a representative property model. The obtained results confirm that the use of these methods leads to the creation of a model that enables a more reality-based view of the uncertainty and imprecise residential environment.

## 1. Introduction

The rapidly expanding urban areas of the world pose a challenge for the 21st century—one that requires new analytic approaches and new sources of data/information.

Real estate plays an extremely important role in the global economy, has ecological significance, and contributes to human health at the same time. It is also a vital ingredient of the urban environment. The measurable indicators such as house price or value are used to design the environment [1]. Due to social aspects, residents choose a place based on two contradictory criteria: on the one hand, people want safety, familiarity, and the avoidance of nuisance; on the other hand, they want grandeur, excitement, and stimuli at the same time [2]. A combination of many factors affects the life and health of individuals and communities [3]. Whether people are healthy or not is very often determined by their circumstances and environment. To a large extent, factors such as where we live, the accessibility and use of health care services, and other locational points of interests all have considerable impact on quality of human life. According to the World Health Organization, houses are the one of most important factors affecting health status [4]. People spend roughly 90% of their time indoors, with most of that time likely being in their home. This emphasizes the potential impact housing can have on a person’s life quality [5].

The valuation of property, both individual and mass, often raises a lot of controversy/doubts, whether due to ambiguous regulations in this area, or the strong emotional relationship of individual investors to the property, which is often their place of work, education, residence, social life, impact on health, and well-being, etc. The assessment/mass appraisal system that valuates the residential properties by taking into account natural and anthropogenic factors strongly reaffirms that health (…) is a fundamental human right (…) the realization of which requires the action of many other social and economic sectors in addition to the health sector [6]. Examples of environmental factors affecting life quality status are often housing-related. Households report “great difficulty” in accessing recreational or green areas. Air quality, noise, and proximity to urban infrastructure can arguably have an important impact on the quality of life. Environmental quality (the price of good health) has become a central tenet for consumer choice in urban locations when deciding on a residential neighborhood. The influencing factors of people’s life quality are mainly analyzed from the perspectives of the geographical environment and individual attributes [7]. The influence of the geographical environment comes from the physically built environment [8] and social environment [9,10]. The aforementioned examples of the hard-to-measure impact of real estate on people’s lives should be reflected in its value and the systems that deliver it (e.g., Automated Valuation Models—AVMs).

The investment perception of real estate is undeniably influenced by its location in space. The elements of the environment that form the property’s surroundings, often seen as priceless assets (due to, among other things, most being public (or common) goods, which implies general accessibility and the inability to exclude anyone from use), are important to take into account in the valuation of real estate as an important element of investment decisions.

According to Canter and Kenny [11] “unless there is an understanding of the role that the physical environment plays in people’s lives it is extremely difficult to know which aspects of that environment to measure and how to argue for the significance of any relationships which are found between the environment and human actions or experience”. In this issue, the main task of residential environment evaluation is to take into account the links between the real estate market and the real estate environment (technical attributes and features of environment of analyzed property) in the value of real estate in order to reflect the actual view of the market and, therefore, improve the decision-making process in the real estate market.

According to Kauko [12], real estate research is still a relatively neglected, trivialized, and under-theorized problem area pushed to the margins of economic and technical disciplines. Practical solutions are still in progress especially from a methodological and numerical point of view. Key in this aspect is the adoption of a holistic approach to determining the value of real estate, and thus also the selection of a representative property for mass evaluation purposes. The determination of a representative property, assuming the homogeneity of features with environment and technical attributes, allows the indirect determination of the value of the environment (its elements) expressed by the value of real estate in homogeneous zones. The approach to real estate as an economic and public good is directly related to and intersects with environmental goods through the use of tools that allow the processing of data that are often imprecise and uncertain and make it possible to reflect the real nature of the real estate market.

Any analysis of the real estate market should take into account that the interdependencies within the market may be purely random and—complicating matters further—in constant flux. However, the rapid development of technology bodes well for our capacity to solve complex and multifaceted problems. Intelligent systems, including soft computing techniques, are becoming more important as the power of computer processing devices increases and their cost is reduced. The need to develop soft computing systems is greater than ever. With the growing awareness of how difficult it is to discern the intricacies of the property market and “artificially” reduce it to a strictly deterministic and essentially perfect phenomenon (the so-called “perfect market”), there is more need than ever to develop soft/fuzzy methods that would be near-optimal, more satisfactory, and more effective than the traditional, “categorical” models.

As such, the aim of the paper is to develop a methodology for identifying a representative residential property model using modern and effective analytical techniques. The paper puts forward soft computing methodologies (including approximate reasoning and functional approximation) and experimental work related to identifying a representative property model that exist in an uncertain and imprecise information environment. Therefore, a hypothesis was formulated: methods based on the human mind’s ability to reason (Rough Set Theory (RST) and Self-Organizing Maps (SOMs)) offer a better representation of the real estate market than classical analytical methods (Ward’s Method) in terms of goodness-of-fit between the model and the market realities (transactional prices). The hypothesis, if proven, would also confirm the following thesis: selecting representative residential properties with the use of methods based on cognitive algorithms (soft computing methods) can lead to the creation of a model that would enable a more reality-based view aimed at residential environment evaluation.

The authors of the current study tested the hypothesis and research thesis on the basis of real estate in general and the real estate market in particular. This example can be successfully compared with other phenomena, given its complex, multifaceted character, as well as its characteristic uncertainty, vagueness, and imprecision of property data and the associated relationships. The future of the environment (natural and anthropogenic) and technical attributes of the property were considered for the selection of a representative property in the selected residential local market. Properly selecting the representative property for real estate valuation purposes is crucial for taxation and urban infrastructure development policy, as well as for the overall quality of life of real estate market participants.

The need to discuss representativity for mass appraisal is due to the fact that the same simplified analytical models are still used to select a representative property, which are intended to provide a view of behavior and trends in the real estate market. There is a need to look for new concepts and solutions tailored to the specifics of the real estate market, the contemporary requirements of real estate market participants, and as an answer to the dynamic development of new technologies.

In accordance with the assumed purpose of the article, and the assumed thesis and hypotheses, the authors propose a solution to support the process of automatic real estate evaluation. The methodology presented in the article for selecting a representative property is based on an iterative process of selecting the optimal model of property (transaction) similarity.

The work is structured as follows. First (Section 1), the authors explain the rationale for seeking new methods and approaches for identifying representative properties (a model) in terms of the residential environment evaluation, that is, the indispensable element of a more realistic representation of an uncertain and vague phenomenon. Section 2 presents an overview of the commonly used definitions of representative property and the relevant solutions (methods). Section 3 and Section 4 delineate the materials and methods used, as well as the methodology used to determine the representative property model. Section 5 presents empirical results obtained on the basis of residential real estate case studies. The verification of the results is presented in Section 6. Finally, Section 7 and Section 8 present the discussion and conclusions, as well as the rationale for further research.

## 2. Literature Review

The approach to determining a representative property for mass valuation purposes is a problem considered by researchers and practitioners conducting real estate market analysis. Real estate in its essence is not just a product, but a kind of integration of the needs met by it (social, economic, environmental) due to its position in space. An appropriate selection of features (technical attributes and features of environment) for the selection of a representative property, as well as appropriate methods and tools, is essential to reflect in the value of a property the extremely important influence of the environment and surroundings in which the property is located. Such an approach is key to maintaining sustainability and reflecting the essence of integrating the environment into the residential real estate market.

### 2.1. Methods and Models for Real Estate Valuation

Market analysis is an indispensable element of environment policy and stewardship. One of the key problems in analyzing the real estate market is the recognition of information and, consequently, attributes (their occurrence, scope, and significance) affecting decision-making in a residential environment.

The problem of insufficient property data or the unavailability of information on the property market increases uncertainty in the analysis. Adding to the complexity, despite the imprecise and fuzzy nature of the investment estate, market actors and theories operate under the assumption that the outputs (such as property values) will be highly accurate and precise.

Market analysis methods (including appraisal-related ones) may incorporate a range of analytical tools or techniques and use different types of models, both in traditional real estate appraisal and in automated mass appraisal. Many have noted the arbitrary and subjective nature of traditional comparative property appraisal methods [13]. An obvious hurdle in mass property valuation relates to the difficulty of correctly defining similarity metrics and measuring property value determinants. According to the IAAO Standard of Mass Appraisal of Real Property [14] “mass appraisal is the process of valuing a group of properties as of a given date and using common data, standardized methods, and statistical testing”.

Real estate valuation is a multi-step process. It should be emphasized that identifying the similarity between properties—one of the key stages in individual and mass valuation—is based on a classification basis, i.e., grouping the properties into relatively uniform (homogeneous) sets. Mass property appraisal requires that representative properties are selected to describe the characteristics of a given type of property in a given (e.g., municipal/communal/district) valuation zone. It is the purpose of the general real estate valuation, in which the values of the real estate are determined according to its characteristics. Including both the technical attributes of the property and the features of environment that play an important role in determining the price/attractiveness of the property.

In summary, the complexity of mass property appraisal usually calls for the use of hybrid models that combine multiple methods (to be used at different stages of the process).

### 2.2. Analysis of Object Similarity—Discussion

Choosing a representative of a wider group of similar objects is a complex issue, with different approaches based on the represented phenomenon and the purpose of analysis. Representativeness is a term used to describe how characteristic a particular item is of the types of goods and services included in a basic heading [15]. Another question to consider is whether a “similar” object equates with a “homogenous” one. The term “homogeneous” refers to a uniform, coherent whole made up of similar elements [16].

Clearly, similarity can be characterized and defined in a number of different ways. Defining and then assessing similarity is a significant challenge. In particular, it requires that similarity criteria be defined as appropriate to the needs of the given study. The authors may suppose, after Walesiak [17], that the less objects differ from each other, the more similar they are. Similarity measurement is most often carried out with the use of grouping, classification or delimitation methods that draw upon various measures of similarity. Makowska [18] posits that objects of a single set should exhibit maximum similarity (the criterion of homogeneity postulate of internal cohesion) and that objects separated across sets should exhibit maximum dissimilarity (the criterion of heterogeneity, postulate of external isolation).

The situation is made all the more difficult if the data and information available are highly generalized, ambiguous, imprecise, and therefore highly uncertain, which is certainly true for the real estate market. In such cases, the methods and technologies should be selected so as to enable a more flexible (relaxed) classification approach.

### 2.3. Representative Property Definitions and Aims

The term “representative property” is predominantly used in the literature in reference to mass appraisal for the purpose of calculating ad valorem property taxes. According to Sawiłow and Akincza [19], representative property is typical property that can be distinguished within a given valuation zone, assuming that price-determining attributes are homogeneous. These attributes relate mainly to locational (environment), physical, technical, and practical features determined for properties within a given homogenous zone. In the next stage, the different property value determinants are weighed, which can be done in various ways: arbitrarily (based on market data) or by using various statistical formulas. Thus, as stated by Makowska [18], one of the assumptions of the commonly used pairwise comparison method is that both the buyer and the seller make decisions that are not only optimal for themselves but also predictable to observers. Therefore, the chosen comparables must meet this criterion, i.e., the transaction relating to the comparable must be representative of the given market (must be carried out by a typical buyer).

The definition of representative property for the aim of Property Prices Indices according to the World Bank and Eurostat is: “a property, or category of properties, that accounts for a significant proportion of the total expenditures within some aggregate, and/or for which the average price change is expected to be close to the average for all properties within the aggregate” [20,21]. According to the Handbook on Residential Property Prices indices [21], at the heart of this concept lies the definition of a “typical property” or a “homogeneous local market”, which serves as a fixed reference point for determining indices. However, Foryś [22] argues that the criterion of similarity is imprecise—the adopted attributes are in many cases influenced by the specific purpose of the acquired information. Due to the nature of the real estate market, there is no final and enduring set of attributes for individual property types that could be used to create future-proof price indices. Consequently, many methods and property price indices are used concurrently [23], differing mostly in their approach to simplifying the attributes of the examined objects to achieve comparability (in order to mitigate the limitations brought on by the changes in property over time, as well as changes in buyers’ preferences).

A representative property is also called for when determining property charges in cases where the property value changes, e.g., due to local development, changes in local zoning plans, or construction of technical infrastructures, as well as when determining compensation, e.g., for the expropriation of private property for public interest, using mass appraisal. A representative property also needs to be derived when determining preferences of property investors or when preparing investment advice. In this case, there are no formal rules for selecting a representative, leaving us with informal assumptions and intuition. It follows that establishing the right methodology for choosing a representative property is a highly challenging and multilayered task.

In order to ensure that the solutions are universal and objective, a detailed literature review was performed on the subject of defining and assessing representativity as a research problem. The earliest formulations of the subject focus on the concept of a representative sample. Generally, a representative sample is one whose structure (in terms of the considered attributes/variables) reflects the structure of the statistical population from which it derives. Often such samples are considered to be the most average, typical of the population [24]. According to Jabkowski [25], a representative survey is a one which is conducted on a sample consisting of individuals who reflect the features of a population. Thus, after the sample is measured, the results can be extrapolated to the entire population at an predetermined confidence level and a known error value. A more detailed definition is provided by Lissowski et al. [26], stating that a representative sample for a given population and variable (or set of variables) is a sample which produces an estimate of the population parameters for the variable (set of variables) within the limits specified by the precision requirements and within the corresponding values for the population. The procedure for randomly selecting representative samples must provide such samples with the probability indicated in the predetermined confidence level.

Representativity has been examined through a formal statistical lens, as well as in more informal contexts. One of the more interesting approaches holds that a representative measurement must include all the values of the considered variable that actually occur in the population [27]. This general statement has been expanded to include the condition that the empirical distributions of variables (or values of estimators) should correspond—within a specified margin—to the actual distributions (or values of parameters) in the population as a whole. Clearly, then, the concept of representativity is ambiguous.

Studies on representatives point to the general conclusion that representativity is a fuzzy concept, non-categorical in nature and result. Jabkowski [25] emphasizes this in his work, by stating: it is therefore not prudent to say that a research sample must be either representative or unrepresentative. Firstly, the degree of representativity depends on the accuracy of the parameter value measurements performed using the sample. A sample may be representative for certain variables, but not others, which Davern [28] concurs with.

Taking into account that the concept of representativity is ambiguous, the authors define their own assumptions and definition of representative property. To summarize this review of the current state of research on the subject, the authors believe that the derivation of the representative property should be done according to the following criteria:

- it cannot be a virtual, non-existent property, in keeping with the theory and principle stating that: “*the strong definition of representativity assumes that a data subset is strictly representative if the response propensities are the same for all units in the sample*” [29,30];
- it is not an absolute and categorical (finite) category of objects, but rather a rough set according to the assumed definition of similarity, as per the principle of: “*It is therefore not prudent to say that a research sample must be either representative or unrepresentative*” [25];
- a representative property has a high likelihood of possessing certain attributes, in accordance with the principle stating that: “*A sample can be considered representative if the empirical values of estimators correspond—within a specified margin—to the actual values of parameters in the population as a whole and if the relationships between variables determined on its basis are true to the actual relationships within the population*” [27];
- the representative property combines two attribute modules: features of environment (natural and anthropogenic), i.e., the surroundings, and technical attributes, i.e., internal ones, as per the criteria for selecting a representative property: “*a representative property is typical property that can be distinguished within a given valuation zone, assuming that price-determining attributes are homogeneous. These attributes relate mainly to locational, physical, technical and practical features determined for properties within a given homogenous zone*” [19];
- when selecting a representative property, the attributes are classified according to their relevance on an ad-hoc basis by measuring the information content in data sets: “*there is no final and enduring set of attributes for individual property types*” [22,23];
- the attributes of the representative property should be selected according to their: “*effect on the value or appeal of the property*” [31,32];
- adopting a raw (unprocessed) data set: “*based on divergent observations treated as vital market information, rather than data errors*” [33].

To summarize, the proposed representative properties’ definition assumes that the set of properties that are price-determining attributes are homogeneous. The homogeneity is defined on the basis of fuzzy/approximate level of similarities of the grouped transactions, and the property representative model is based on existing transactions—without averaging the results and adjusting the attribute weights. This avoids the oversimplification of the substantive error of fitting the valued property to the representative property model (that is, in fact, contrary to the state-of-the-art).

## 3. Data

A key part of determining a representative property model is the selection of attributes. This stage consists of a twofold analysis: of the features of the property’s environment (anthropogenic and natural) and technical attributes. The study was conducted using spatial and physical data for real estate in Gdynia city. Gdynia (Northern Poland located nearly the Baltic sea—geographic coordinates for the centre Φ = 54°31′08″ N, Λ = 18°31′54″ E) is continuously developing city with a very good economic condition perspective. The Tri-City is one of largest housing market in Poland, where approximately 8.5 thousand apartments are sold annually. Gdynia is very highly ranked for its quality of life and environment health and because it is a desirable place for residential investment. The city was chosen for the study due to it being a highly developed agglomeration with diverse and numerous spatial features that may serve as a suitably appropriate case study.

### 3.1. Features of Environment

The different types of variables in different scales such as features of environment can affect real estate analysis results. According to Soltani et al. [34] understanding real estate prices and those key characteristics that determine property values is an important factor in planning our cities and setting housing policies. Real estate market conditions (reflected in property values) can differ significantly from location (features of environment) [35]. The importance of real estate location features in the context of property prices and values has been studied by many researchers [36,37,38,39,40]. Features of the environment determine the preferences and behaviors of buyers [35]; therefore, it is necessary to include them in the analyzes of representativeness on the real estate market. In order to describe the examined area, a number of characteristics that provided information on real estate location and attractiveness were investigated via an extensive literature review [41,42,43]. It should be noted that location as an attribute of real estate consists of multiple sub-attributes, which should be analyzed in synergy. One of the factors mentioned quite often in the literature is related to the proximity and accessibility of facilities and services [44,45,46]. The facilities include education centers (e.g., universities, primary schools, secondary schools, kindergartens, etc.), health care (e.g., hospitals, dentists, doctors, etc.), shopping centers (e.g., convenience, beauty shops, bakeries, fast foods, etc.). The reason for their significance is usually connected with the suitability of the neighborhood, the need and frequency of their use, and the time required for it. Another cited characteristic is the environmental conditions and the quality of elements reflecting them and its constituents [47,48]. The elements that seem to have the biggest influence on the residential property purchase decision include water, air or noise pollution, and the availability of green spaces. The environmental conditions that are also taken into consideration include the risk of natural hazards (e.g., floods, earthquakes). Another factor in residential property purchase decision-making relates to transport availability for the property [43,49,50,51,52,53]. The feature is usually interpreted as spatial accessibility determined by public transport elements existence (bus stops, rail, metro). Distance from central business districts or public facilities can also be connected with another feature, usually measured as commuting time. Finally, the location characteristics often described in the literature also include neighborhood aesthetics and social/economic background [54,55,56,57]. Even though the factors might seem completely different, they are usually strongly correlated. The dominant ethnicity, language, religion, family unit size, education level, etc. usually influence the surrounding aesthetics.

The most representative factors providing information on residential environment attractiveness were described with the following attributes (Table 1) [58].

### 3.2. Technical Attributes of Real Estate Related to General Property Condition

Real estate is described in terms of its environment, as well as its specific technical and functional attributes. The real estate transaction database used for the analysis contains data on 2359 transactions across the period of 2017–2019. The properties were characterized using five attributes (Table 2), which had a major impact on their value (as determined by mass appraisal) in the analyzed market across spatially homogenous areas. The selection resulted from the common part between the analyzed valuation reports and full coverage of available attributes in the database.

The domains of property features are presented in a way that enable the simultaneous processing of quantitative and qualitative data.

## 4. Methodology

**Scheme 1:**
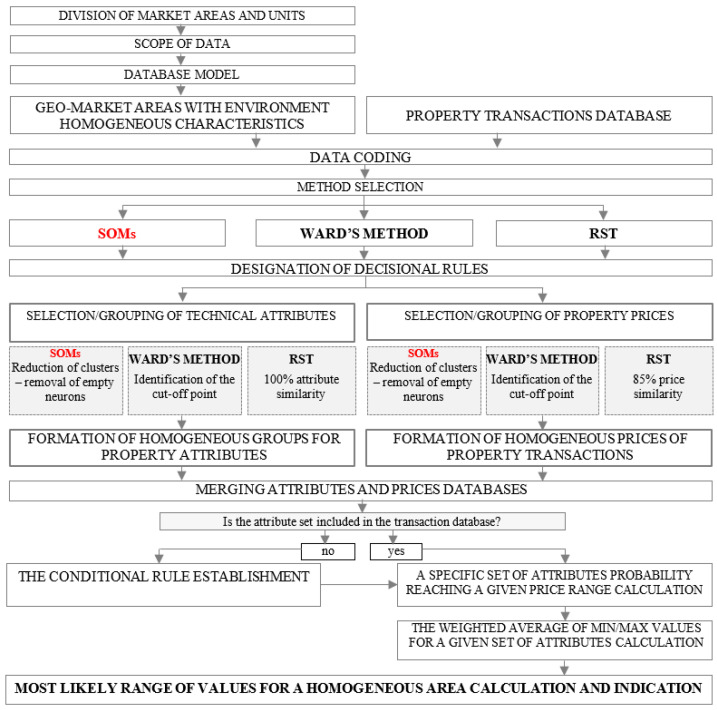
Procedure used to derive the representative property model (source: own elaboration).

The procedure of the representative property model indication is based on the main stages presented below (Scheme 1).

### 4.1. Soft-Computing-Based Methodology for Real Estate Valuation

The assumption is that a representative property model should be designated from a homogeneous group of properties. At this stage, the issue is: how to determine property similarity described with many features based on one synthetic variable that describes properties (such as structures) in a multidimensional area. The real estate market landscape is shaped by processes and relationships that we can predict with some probability, but also by events that are random, obscure, and difficult to predict, of random nature. Such events include, but are not limited to: volatility of property attributes, conflicting information, inconsistent access to information, impaired judgment of real estate market actors, uncertainty of systemic structures and functions, and emotionally charged approach to transactions by actors [59,60]. Traditional calculation methods require a thorough understanding of the problem and the relationship between variables, which makes it impossible to conduct reliable analyses in complex and fragmented spaces. Soft computing is an array of bioinspired computation techniques used to process large sets of data [61].

Soft computing is an approach to computing which parallels the remarkable ability of the human mind to reason and learn in an environment of uncertainty and imprecision. Unlike hard computing, soft computing is tolerant of imprecision, uncertainty, partial truth, and approximations [62]. To assess the potential of soft computing approaches, the authors used methods based on Self-Organizing Maps (SOMs) and Rough Set Theory (RST). The selected methods such as SOMs and RST competitive learning were chosen due to their character that “understand” the specificity of the property market data that are:

- flexibility taking into account the quantity of the dataset,
- high efficiency in case of optimized clustering,
- unsupervised learning solution,
- flexibility in data dimension reduction,
- using raw input data without a priori definition of relationships.

On the other hand, cluster analysis (Ward’s Method) was used to assess the quality of results derived using a traditional solution (commonly used for market analysis) for comparison.

### 4.2. Rough Set Theory

The elaboration of a representative property model was done using RST based on Fuzzy Logic and Entropy Theory [58]. The use of this methodology was motivated by the specificity of the analyzed information such as: overload and unavailability, the heteroscedasticity of property geolocation, and the rationality of problem solving related to residential property analyses.

The RST [63,64,65], based on discrete mathematics, may be applied with little data without a causal mathematical relationship. A more flexible way to deal with the indiscernibility relation was obtained thanks to the application of the value tolerance relation [53] to the classical RST (based on a crisp indiscernibility relation).

In order to verify the similarity (indiscernibility) of particular attributes, the “valued tolerance relation” (VTR)] was applied (Equation (1)):

(1) $$R_{j}\left(x,y\right)=\frac{\max\left(0;\min\left(c_{j}\left(x\right),{\;c}_{j}\left(y\right)\right)+k-\max\left(c_{j}\left(x\right),{\;c}_{j}\left(y\right)\right)\right)\;}{k},$$

where: $R_{j}$(x, y)—relation between objects with a result of membership function [0, 1]; (x, y)—identifier of unit; $c_{j}$—function of the j attribute selection from given property; $c_{j}\left(x\right)$, $c_{j}\left(y\right)$—homogenous attributes of the analyzed property; j—number of attributes; k—threshold for the homogenous attributes set, which, which allows objects to be considered indiscernible despite not having identical values—a standard deviation for data on a ratio scale and one interval for data on a discrete scale.

Whereas the $W_{x}$ is the set of all similar (indiscernible) properties to x, and its description fulfills the following rule (Equations (2) and (3)):

(2) $$p\in W_{x}⟺T\left(R\left(x,p\right)\right)=\mathrm{TRUE}$$

(3) $$\;P=\left\{p_{1},p_{2}\ldots p_{m}\right\};m\;–\;\mathrm{number}\;\mathrm{of}\;\mathrm{properties},\;m\geq 1,$$

The set of $W_{x}$ (set of similar properties to x) consists only of those $p_{i}$ from the set P set, that satisfy the condition defined as a function of the tolerance T, where: $p_{i}$—property/object that is a prospect for the given homogeneous group considered as a rule.

In order to take into account a large variation in data, classes of indiscernibility were determined on the basis of the assumed percentage of similarity (pc). In this stage, Equation (4) on the toleration function is as follows:

(4) $$T\;\left(R\right)=\mathrm{TRUE}\;\Leftrightarrow \;R>\mathrm{pc}\;\max{\mathrm{IND}}_{i}\left(B,d\right)$$

where: ${\mathrm{IND}}_{i}\left(B,d\right)$—d attribute in the set of B attributes that are considered indiscernible; i—number of indiscernible set of units.

The maximal credible and similar sets of decisional rules were performed by assigning a repetitive element of the decisional rule to its particular “class of rules ($d_{i}$)” as follows: y belongs to the $d_{i}$ decision class where $R_{\min}\left(x,y\right)$ for $d_{i}$ is maximal, which means the maximization of the least similarity with one pair of objects ($x_{i}$ and $y_{i}$)—Equation (5):

(5) $$\mu_{I}\left(p_{i}\right)=\min_{x\in S_{p_{i}}}\left(\max R_{B}\left(x,{\;p}_{i}\right)\right)$$

where: $\mu_{I}$—first similarities condition; $S_{\mathrm{pi}}$—objects similar to the conditional part of the analyzed rule.

In case of repetitive results’ obtainment, the rule of maximum of the sum from $R_{j}$ proving maximal indiscernibility of the unit and the rule is applied (Equation (6)):

(6) $$\mu_{\mathrm{II}}\left(p_{i}\right)=\max_{x\in S_{p_{i}}}\sum_{j=1}^{n}R_{j}\left(x,{\;p}_{i}\right)$$

where: $\mu_{\mathrm{II}}$—second similarities condition.

The decisive assessment of the created rules involves comparing the percentage of the credibility that is calculated within Equation (7):

(7) $${\mathrm{Cr}}_{i}=\frac{\left|\left\{P|P\in g_{i}|P_{N}=1\right\}\right|}{\left|g_{i}\right|}\;;\;{\;P}_{n}=\left\{p_{n_{1}},p_{n_{2}}\ldots {\;p}_{n_{m}}\right\}$$

where: Cr—set of credibility of particular groups; $\;\mathrm{Cr}=\left\{c_{r_{1}},c_{r_{2}}\ldots {\;c}_{r_{s}}\right\}$; $P_{n}$—set of repetitions of particular p in G; $\;G=\left\{g_{1},g_{2},\ldots g_{s}\right\}$; G—set of p groups; s—number of proposed groups, s≥1; $g_{u}=\left\{p_{u_{1}},p_{u_{1}}\ldots p_{u_{z}}\right\}$; u-th group consisting of z units from set P,  z≥1.

### 4.3. Self-Organizing Maps

The SOMs are a type of Neural Network used to generalize a description of collected observations. It was developed by Teuvo Kohonen [66] and is based on unsupervised learning. One of its main applications is in reducing the dimensionality of data vectors by representing their subsets in 2D space. The subsets are matched to a set group of M nodes/units (which are usually defined on a rectangular grid with a set number of columns—x and rows—y). Each node of the grid has a vector of weight (w) values, estimated during the learning process, and is dimensionally/semantically consistent with the input vectors. This iterative processing/clustering can help interpret phenomena and determine their dominant features.

The iterative process is preceded by seeding the weights of all M nodes with random values from the domain of acceptable values (set according to input data) or values of randomly selected input vectors. All of the successive iterations solve for the closest M node (BMU—best-matching unit) for each input vector $x_{i}$. The distance between the BMU and the $x_{i}$ is expressed as parameter b (Equation (8)):

(8) $$b=\|w_{\mathrm{BMU}}-x_{i}\|$$

The values of b are used to adjust the weight values of the BMU and the neighboring nodes in M. The adjustment of weights $w_{l}$ of a given node l neighboring N is expressed as (Equation (9)):

(9) $$w_{l}=w_{l}+\alpha h\left(b,N,l\right){\;\Delta }_{i,l}$$

where: ∝—learning rate; $\Delta_{i,l}=w_{l}-x_{i}$; h—loss function defined as (Equation (10)):

(10) $$h=\{\begin{array}{l} 1\;for\;l\epsilon \;N\left(r,b\right)\\ 0\;for\;other\;cases \end{array}$$

where: r—the radius of acceptable distance for node modification from the BMU initiated with value $r=\sqrt{x^{2}+y^{2}}$ and decreased with each iteration by value $\frac{\sqrt{x^{2}+y^{2}}}{m_{i}}$, where: $m_{i}$—the predetermined maximum of iterations for network learning.

The process of node grid plotting should be controlled for effectiveness and quality of matching with individual input vectors $x_{i}$. This is usually done via the Quantization Error (QE), which measures the average distance between input data vectors and their respective nodes (weights).

This is the basic quality measure for evaluating self-organizing, defined by the formula (Equation (11)):

(11) $$\mathrm{QE}=\frac{1}{n}\sum_{i=1}^{n}\|\Phi \left(x_{i}\right)-x_{i}\|$$

where: n—number of input vectors x; $x_{i}$—i-th input vector with size d; $\Phi \left(x_{i}\right)$—the function mapping the input vector to the respective node of grid M.

Monitoring the QE during network learning may also reveal the final iteration (before iteration mi) of the QE reaching the target value. It may also detect situations where QE does not change during the learning process or changes so little in numerical terms that further learning would be unproductive. SOMs are designed to cluster sets of difficult-to-discern data, therefore, M size must not be set a priori, as it is not an unambiguously or intuitively solvable problem. With that in mind, the authors set the size of M using an empirical approach. In principle, QE should be expected to decrease as the M size increases. With a large enough M, each $x_{i}$ can be matched with its own separate M node, converging all $\left\|\Phi \left(x_{i}\right)-x_{i}\right\|$ values to zero. However, the resultant clusters of vectors x will be zero- or single-object sets. Therefore, the second parameter controlled during the iterative processing was based on the M size to minimize the number of null clusters. An analysis of grids with different w and h sizes—where w={2,3,…,20}, h ={2,3,…,20}—showed that an M of 10 × 10 is the optimal choice for this set of observation vectors.

The attributes were grouped in 5D space (location on the floor, number of rooms, usable area, building age, building materials) based on their similarity using Kohonen’s Neural Networks. The individual attribute states/values were clustered using the Kohonen method, which is a very effective tool for grouping similar objects, using SOMs. The authors decided to use synergistic representative attributes (and thus reduce the size of the Kohonen network model), as this method produces a friendly/usable/simple representation of complex data (a two-dimensional topological map of the input data forming similar clusters). The choice was also dictated by the nature of the input—unclustered data with high variability of the phenomenon, complex internal structure, and no decisional variable.

### 4.4. Ward’s Method

In the literature on the subject, various systematics of grouping methods are proposed. Ward’s Method, as a major agglomerative hierarchical method, is one of the most popular methods used in object grouping problems [67,68,69,70].

In order to identify groups of homogeneous properties, a cluster analysis was performed using a hierarchical algorithm (Ward’s Method), which produced a dendrographic hierarchy of the analyzed set. The hierarchical cluster analysis provided a dendrogram that plots the hierarchical structure of the object set in the order of decreasing similarity.

A database of real estate transactions was fed into the algorithm. For the first step of the analysis, the properties were grouped according to their similarity in terms of aggregates (sets) of property attributes. The applied procedures steps were as follows [58]:

- Step 1: selecting the minimum distant pair of clusters (Equation (12)):

(12) $$d_{\mathrm{pq}}=\underset{i,j}{\min}\left\{d_{\mathrm{ij}}=d\left(\Omega_{i},\Omega_{j}\right)\right\},p<q,$$

where: d—distance between the clusters p i q, $\Omega =\left\{O_{1},{\;O}_{2},\;\ldots ,{\;O}_{N}\right\}\;$—classification set of objects, N- quantity of objects, j=1, …, N.

- Step 2: merging clusters $\Omega_{p}$ and $\Omega_{q}$ into one p (Equation (13)):

(13) $$\Omega_{p}=\Omega_{p}\cup \Omega_{q}$$

- Step 3: dimension changes of matrix $D_{q}$ to $N_{1}$),
- Step 4: the distance $d_{\mathrm{pj}}$ (between “new” cluster $\Omega_{p}$ and the other clusters) calculation; a column/row p replacement on $d_{\mathrm{pj}}$ value,
- Return to step 1: procedure finish when one cluster remains.

At each stage, from among all possible pairs of clusters, the one is selected that as a result of combining gives a focus with minimal differentiation [71]. The criteria for grouping the units in this case is the minimum of differentiation of the vectors of the features that make up the cluster in relation to the mean values in these sets. The measure of the diversity of the cluster in relation to the mean values is ESS (Error Sum of Squares), also known as the error of the sum of squares. This method consists in enlarging the clusters in such a way as to ensure the smallest increase in variance for a given iteration (Equation (14)).

(14) $$\mathrm{ESS}=\sum_{i=1}^{k}\|x_{i}-\bar{x}\|$$

where: $x_{i}$—value of the variable which is the segmentation criterion for the i-th object; k—number of objects in a cluster.

Ward’s Method is the classic hierarchical cluster analysis method for grouping data. Using Ward’s Method, “natural” aggregates can be detected in a set. Unlike other methods, it uses analysis of variance to estimate the distance between clusters.

## 5. Procedure for Determining the Model of the Representative Property

### 5.1. Detection of Homogeneous Areas—HO-MAR

**Figure 1:**
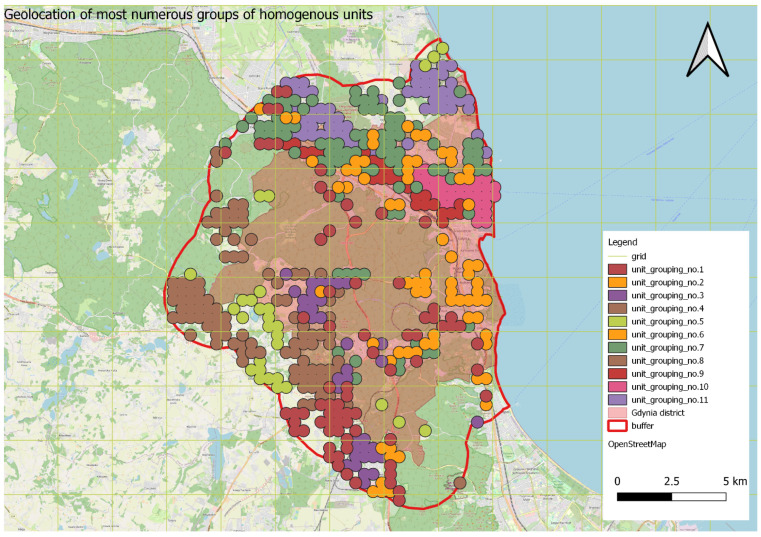
Geolocation of most numerous groups of the homogenous units. Source: based on Renigier-Biłozor, et al. [58].

The representative property model is derived from the area of homogeneous features of environment. The HO-MAR process for detecting homogeneous area has been described in detail by Renigier-Biłozor et al. [58]. The elaborated methodology was based on Entropy Theory, RST, Fuzzy Logic, and geoprocessing activities (Gauss filter, geocoding and reverse geocoding, tessellation model with mutual spatial overlapping). The mentioned procedures produced 166 homogenous areas. The geolocation of the most numerous groups is presented in Figure 1.

A rectangular mesh with a grid element size 2.5 × 2.5 km was added on the map. The homogenous areas were predominately continuous areas (provided from neighboring unit aggregation). In the next step of the process, the isolated homogenous areas of the real estate market were used to identify representative properties.

### 5.2. Methodology—Selection of Representative Residential Property Model

In order to verify the research hypothesis, a methodology was developed to determine representative properties using three methods: RST, SOMs, and Ward’s Method (W).

**Figure 2:**
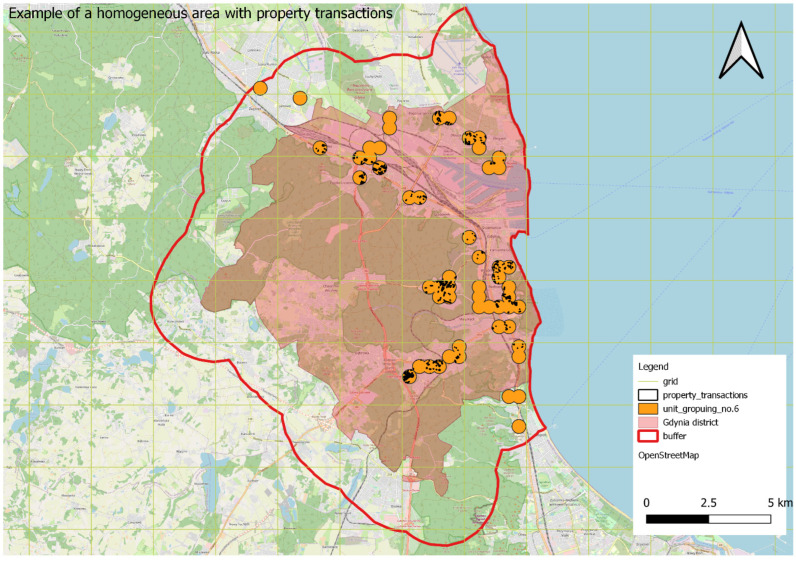
Example of the homogeneous area with property transactions. Source: own elaboration based on Renigier-Biłozor, et al. [58].

In the first phase of the procedure, transactions of properties characterized by five technical attributes (5D vectors) were grouped separately in each homogenous area (see Section 5.1). For a detailed explanation of how representative property models were derived using the different methods, the respective results were illustrated using the example of an isolated spatial homogenous area (in orange) and its market-traded properties (Figure 2).

#### 5.2.1. Rough-Set-Theory-Based Model

During the first step, the RST algorithm produced 140 homogeneous groups of property attributes (RST.Attr.) (Table 3). The full dataset is available in Supplementary Table S1. The groups included anywhere from 26 to 1 transactions. The grouping was performed assuming 100% similarity between objects within a group.

In the second step, the properties were grouped according to their transaction price (Table 4). The full dataset is available in Supplementary Table S2. The authors adopted a indiscernibility threshold (k) equal to the standard derivation in the transactional price set. The internal similarity of the homogeneous transaction price grouping was assumed at 85%. The clustering identified 200 hundred price grouping (RST.Price.) of 1 to 10 property transactions, with the difference between the largest and smallest transaction in the group averaging 65 PLN/m^2^.

The price ranges for individual homogeneous price clusters were determined by the transaction prices included within the given group.

In the third step, the appropriate aggregates (sets) of technical attributes within the homogeneous price grouping (RST.Price.) were identified by merging two databases, matching the individual RST.Attr. attribute groupings to the corresponding price range (Table 5). The full dataset is available in Supplementary Table S3.

The resultant table shows which property attribute aggregates were matched with which price ranges. As seen in Table 5, the individual RST.Attr. attribute groupings could be matched with multiple RST.Price. price groupings due to the nature of the real estate market.

#### 5.2.2. Self-Organizing-Map-Based Model

In the first step, the objects were clustered by measuring the Euclidean Distance between individual (sets of) property attributes to the winner neurons (the values closest to the individual objects in the group). This produced a 10 × 10 starting matrix, with the target minimal QE for dataset training being 0.00937 (validation dataset 0.01437, testing data set 0.01885). Due to the emergence of empty matrix components, the clusters were reduced by merging, producing 55 homogeneous groups (SOM.Attr.) (Supplementary Table S4). The size of the groups ranged from 38 to 1 objects.

The second step produced 73 homogeneous price groups (SOM.Price.) (with the target QE minimization for data set training being 0.00012 (validation data set 0.00013, testing data set 0.00164)) of 78 to 1 objects per group and an average price spread of 359 PLN/m^2^ (Supplementary Table S5).

In the third step, the two databases were merged (see Section 5.2.1), matching the individual SOM.Attr. attribute groupings to the corresponding price range (Supplementary Table S6).

The resultant table shows which SOM.Attr. property attribute aggregates were matched with which SOM.Price. price ranges.

#### 5.2.3. Ward’s-Method-Based Model

**Figure 3:**
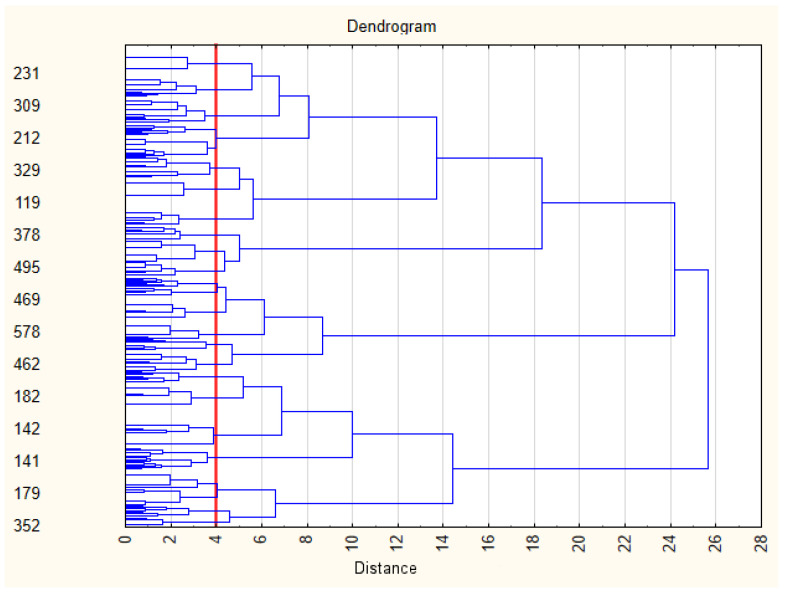
Dendrogram. Source: own elaboration.

In the first step, the objects were clustered by analyzing the variance of distances between individual aggregates (sets) of property attributes. The number of clusters was determined by the cut-off, where multiple clusters formed at approximately the same bond distance were identified. Cut-off 4 was set on the basis of the agglomeration schedule graph which shows the distances of bonds in relation to the subsequent stages of the bonding process (Figure 3).

As the results show, 24 homogeneous groups (W.Attr.) were created (Supplementary Table S7). The size of the clusters ranged from 49 to 11 objects.

The second step produced 21 homogeneous price groups (W.Price.) (cut-off = 0.5) of 85 to 1 objects per group and an average price spread of 538 PLN/$m^{2}$ (Supplementary Table S8).

In the third step, the two databases were merged (see Section 5.2.1) (Supplementary Table S9).

The resultant table shows which W.Attr. property attribute aggregates were matched with which W.Price. price ranges.

## 6. Verification of Models Outputs Versus Transactional Prices

Verification was done using 70 benchmark transactions which were not included in the validation dataset. As before, the verification methodology is illustrated using a selected example.

For the first step of output verification, the authors calculated the empirical percentage of uniqueness (${\mathrm{EP}}_{i,j},$ where i—particular attribute set grouping, j—particular price grouping) of a given homogeneous attribute grouping appearing within the different price ranges (Equation (15)).

(15) $${\mathrm{EP}}_{i,j}=\frac{n_{i,j}}{\sum_{x=1}^{y}n_{i,x}}*100\%$$

where: y—number of price groupings; $n_{i,j}$—occurrence number of particular attribute grouping (i) in particular price grouping (j); $N_{i}$—occurrence number of particular attribute set groupings.

The results are presented in Table 6.

The following equations, Equations (16) and (17), were used to estimate the min/max price range (V) for a given property attribute set:

(16) $$V_{\min}=\sum \left(P_{\min}{*\mathrm{EP}}_{i}\right)$$

(17) $$V_{\max}=\sum \left(P_{\max}{*\mathrm{EP}}_{i}\right),$$

Thus, value ranges were calculated for the respective property attribute sets (Table 7).

In the next step of the analysis, the outputs from the different models (RST, SOM, W) were checked for accuracy with regard to the actual market. To assess the performance of the models, the value ranges produced for the assorted attribute sets were compared against an actual datum of the market, i.e., transaction prices. The methodology for evaluating the models in terms of their accuracy to the market was illustrated using a selected example: a property (no. 32) with known technical attributes and transaction price (located in an area homogenous in terms of location characteristics) was pulled from the benchmark dataset. The authors screened the homogeneous groupings of property attributes (obtained using the three models) and selected groups that matched the examined property in terms of attributes (RST.Attr.23, SOM.Attr.42, W.Attr.23). Model accuracy was measured by means of the distance (D) between transactional price ($P_{t}$) of the benchmark property and the value range ($V_{\min}$, $V_{\max}$) calculated using Equations (16) and (17). The distances between the transaction price and the value range were calculated from the following (Equation (18)):

(18) $$D\left(P_{t},\;(V_{\min},\;V_{\max})\right)=\{\begin{array}{l} 0\;for\;V_{\min}<Pt<V_{\max}\\ V_{\min}\;–\;P_{t}\;for\;P_{t}<V_{\min}\;\\ \left|V_{\max}\;–{\;P}_{t}\right|\;for\;P_{t}>V_{\max} \end{array}$$

Thus, distances were calculated between the individual transaction prices and the value ranges obtained for each of the three models (Table 8).

The value ranges produced by the SOM model were the closest to the benchmark property transaction price.

All properties in the benchmark database (a total of 70 benchmark properties with known physical/utilitarian properties and transaction prices) were input into the verification procedure outlined above. The distances between transaction prices and their respective attribute set value ranges were calculated for each benchmark property using Equation (18). Thus, the authors derived the average distances for all properties in the benchmark database (Table 9).

By using the verification methodology outlined above for all 70 benchmark properties, the authors calculated the average distances of transaction prices to the calculated value ranges (Table 9). The values calculated using the SOMs model were statistically closest in terms of distance (D = 199.13 PLN), meaning that they best mirrored the actual realities of the market. The results obtained using RST were similar (close) to those provided by SOMs (D = 278.23 PLN). Out of all the models, the average values produced by the hierarchical cluster analysis (Ward’s Method) were the most statistically divergent from the actual transaction prices (D = 749.56 PLN). As such, the SOMs model proved to be the most accurate and true to the actual market.

## 7. Discussion

A representative property is not so much a property with specified features and value, but more of a model of value for a selected homogenous area (in terms of features of property’s environment and technical attributes). When taking the traditional view of a representative property (which is an actual or abstract property with specific features and value), there is a risk of generating errors at the stage of comparing a valued property with a representative. If the representative property is approached as a model of value, the valued property (which has been “qualified” into a relevant homogenous group after prior analysis) needs only to be fed into the appraisal model to arrive at its value. This approach reflects the individual nature of local markets/sub-markets, where the significance of a given feature may vary from ‘region’ to ‘region’. For example, quality housing may not be affected much by a first floor location—or, in another example, a high-rise position may be that much more valuable in a desirable location. The presented approach points to the following interpretation of similarity in terms of properties: alternative goods, competitive for a potential buyer, are understood to be two similar properties in similar markets, even though valuation principles would dictate that they should be characterized by the same location.

The authors of the article, based on the findings of the related studies, which confirm the effectiveness of Artificial Neural Networks (ANN) in solving problems where object function is difficult to define, decided to investigate the potential of Self Organizing Map (e.g., Kohonen’s Neural Network) in the task of searching for representativeness (as a key step in mass valuation). The aim of the article was not only to test the applicability of non-traditional methods in this field, but also to promote a different approach to valuation while fully accepting the complexity of market data and their “imperfection”. Thus, the authors’ main emphasis was on the methodological and logical soundness of the models used to select representative property. The proposed methodology (see Section 2.3) was designated to address key weaknesses in market analysis and data, and thus produce more reliable results. The phrase “more reliable results” in relation to the real estate market refers, on the one hand, to reflecting the particulars of the market “as-is”, and on the other—to allowing property value to be taken as a range. The results produced across the three methods provided an opportunity to compare three completely different ideological and mathematical approaches to choosing representative properties, as verified against the obtained value of the transaction property.

The calculated accuracy of the models, expressed as the differences in real estate price and value, indicate that the NN-based methodology gave the best results in terms of being true to the actual market data, confirming the thesis and proving the hypothesis from the mathematical point of view.

However, the aim of the article was not only to test the applicability of non-traditional methods to this field, but also to promote a different approach to valuation while fully accepting the complexity of market data and their “imperfection”. Thus, the main emphasis was on the methodological and logical soundness of the models used to select residential representative property. The proposed approach can be actualized thanks to soft computing methods, which are flexible and can mimic the human reactions so essential in real estate decision-making systems crucial for environment policy and stewardship. The main differences (and also the novelty) between the study proposal and the currently existing and commonly used solutions include the following assumptions:

- division of the analyses consist of twofold aspects: features of property’s environment and technical attributes;
- representative property is not an artificial phenomenon—virtual, non-existent property;
- taking into account the specificity of the real estate market not in a categorical approach, but as a fuzzy soft phenomena;
- the principle of raw data preprocessing limitation implementation;
- market area/scope is not defined a priori as a precise boundary;
- property market zone is not defined by the only spatial relations/proximity;
- homogeneous comparable spatial units topology defines complex irregular disjoint or overlapping polygons (allowing existence of spatial holes), thus, homogeneous groups of properties do not require to be located in immediate vicinity;
- the unit value of the representative property is not a point but a range of values.

The authors also stress that every mathematical method as a simplified representation of the world has its strengths and weaknesses. The major practical advantages and disadvantages of each method incorporated into the analysis are presented below (Table 10).

The authors believe that, when combined with the methodological approach, each of these models can be a valuable alternative depending on the quality of data and the real estate market conditions.

## 8. Conclusions

Soft computing is an integral component of methodology for modeling real issues and phenomena which are difficult—or even impossible—to model with traditional approaches (based on mathematical and statistical canon). Soft computing methods strive to adapt and parallel the functioning of the human mind, which processes input data against a space of expected results or assessments. Robust modeling of processes not expressible in purely mathematical terms can be made possible by increasing tolerance for parameters: approximation, uncertainty, imprecision, and partial truth in order to achieve close resemblance with human-like decision-making. This increased tolerance not only makes such modeling feasible, but also provides robust results at low processing costs.

The proposed methodology provides satisfactory results that account for the complexity and “imperfection” of the real estate market. The proposed “soft value” of an appraised property, expressed as a spectrum, may be considered controversial, but in some applications—including mass valuation or determination of investment portfolios—its less categorical and rigorous nature may be more true-to-life in terms of market data and analyses than discrete scalar values. Moreover, the methodology can fully accommodate the characteristics and specifics of the available market data and the shortcomings of real estate markets as “precision systems” to be rigorously analyzed. Hence, the process of selecting a representative property model was divided into two main steps: a locational/spatial analysis and a utilitarian/physical analysis. A statistical verification of the models showed that the Neural Network methods were the closest to reproducing the realities of the market. However, this does not preclude the use of the other methods as alternatives, whose strengths and weaknesses have been presented in the discussion.

In summary, the primary contribution of this study is the development of a procedure based on a quasi-optimal logical/mathematical model, which uses the actual conditions of the real estate market as its main basis. This methodology is a response to the ubiquitous practice of constructing oversimplified models that run a high risk of misrepresenting the analyzed phenomenon. The value of a property as an object in space should take into account the influence of features of environment due to their undeniable impact on the health and quality of life of real estate market participants. Up-to-date, accurate, and complete knowledge of properties and their importance in shaping the residential environment is required for decision-making related to property valuation, tax assessment, spatial policy, and the overall well-being of property market participants. An important characteristic of a good urban residential environment is that it offers residents a choice of all the types of residential environment they need.

## Figures and Tables

**Table 1:** Features of environment. Source: Renigier-Biłozor, et al. [58].

Layer	Content	Form	Quantity
Buildings	buildings	polygon	51,203
Commercial	bakery, bar, beauty shop, beverages, bicycle shop, bookshop, butcher, café, car dealership, car wash, chemist, clothes, convenience, fast food, florist, food court, furniture shop, garden center, gift shop, greengrocer, guesthouse, hairdresser, hostel, hotel, hunting stand, jeweler, laundry, mobile phone shop, newsagent, outdoor shop, pharmacy, pub, restaurant, shoe shop, sports shop, stationery, supermarket, toy shop, travel agent	points	1749
Public services	community center, fire station, police, post office, town hall, nursing home, bank	points	148
Health care	hospital, dentist, doctor, veterinary, optician	points	212
Education	college, kindergarten, school	points	50
Culture	arts center, artwork, cinema, library, museum, theatre	points	48
Recreation	playground, swimming pool, sports center	points	79
Roads	cycleway, living street, primary, primary link, residential, secondary, secondary link, service, step, tertiary, tertiary link, track, trunk, trunk link, unclassified	line	15,880
Green spaces	farmland, farmyard, forest, nature reserve, allotment	polygon	883
Rail	rail	line	737
Coastline	beach	polygon	14
Parking space	parking, parking bicycle, parking multistorey, parking underground	polygon	848
Bus stops	bus stop	points	737
Airport	airport	polygon	1
Railway station	railway halt, railway station	points	14
Water	dock, reservoir, riverbank, water, wetland	polygon	460

**Table 2:** Technical attributes in property database. Source: own elaboration.

No. of Transaction	Location on the Floor	No. of Rooms	Usable Area of Real Estate	Construction Year	Building Materials
2359	1: ground floor (for construction year to 2000) 2: 3rd floor and above (for construction year to 2000) 3: 1st and 2nd floor (for construction year to 2000) and all of floors in the modern buildings (the construction year after 2000)	the number of rooms in the apartment	1: <25 m^2^ 2: 25–40 m^2^ 3: 40–55 m^2^ 4: 55–70 m^2^ 5: 70–90 m^2^ 6: > 90 m^2^	1: <1965 2: 1965–1995 3: 1995–2010 4: >2010	1: concrete slab 2: no concrete slab

**Table 3:** Example of RST.Attr. groupings of homogeneous property attributes sets within a selected homogeneous area. Source: own elaboration.

RST.Attr.No. *	Transaction Number																									
RST.Attr.1	24	30	31	38	47	54	55	85	91	119	136	170	171	184	191	200	201	216	256	333	415	419	421	441	479	519
RST.Attr.2	0	26	37	43	45	46	48	49	50	75	90	116	118	133	139	183	206	209	210	417	424	445	482	513	-	-
RST.Attr.3	67	72	89	129	144	146	147	159	181	186	190	192	194	198	203	205	207	229	230	538	564	583	610	-	-	-
RST.Attr.4	69	140	202	237	262	263	264	334	336	337	339	437	439	440	486	488	525	536	573	577	-	-	-	-	-	-
…																										
RST.Attr.140	599	-	-	-	-	-	-	-	-	-	-	-	-	-	-	-	-	-	-	-	-	-	-	-	-	-

* No. of homogeneous group (property attributes set).

**Table 4:** Example of RST.Price. groupings of homogeneous prices of property transactions within a selected homogeneous area. Source: own elaboration.

RST.Price.No. *	Transaction Number			Transaction Price [PLN]	
				Min	Max
RST.Price.1	444	113	189	327.87	1685.39
RST.Price.2	597	437	596	2500.00	2500.00
RST.Price.3	124	274	482	2790.70	2872.34
RST.Price.4	230	229	-	3000.00	3000.00
…					
RST.Price.200	220	-	-	30,794.12	30,794.12

* No. of homogeneous group (property transaction prices).

**Table 5:** Example of matching RST.Attr. groupings of homogeneous property attributes with the RST.Price. homogeneous price groupings within a selected homogeneous area. Source: own elaboration.

RST.Price. No	RST.Attr.No			Transaction Price [PLN]	
				Min	Max
RST.Price.1	RST.Attr.14	RST.Attr.68	RST.Attr.42	327.87	1685.39
RST.Price.2	RST.Attr.59	RST.Attr.4	RST.Attr.59	2500.00	2500.00
RST.Price.3	RST.Attr.59.	RST.Attr.11.	RST.Attr.2.	2790.70	2872.34
RST.Price.4	RST.Attr.3.	RST.Attr.3.	-	3000.00	3000.00
…					
RST.Price.200	RST.Attr.67	-	-	30,794.12	30,794.12

**Table 6:** Empirical probability of a given homogenous attribute grouping occurring within the different price groups, illustrated using an example homogenous area. Source: own elaboration.

RST.Attr.23.				SOM.Attr.42.				W.Attr.23.			
RST.Price.No.	Price Range [PLN]		EP	SOM.Price.No.	Price Range [PLN]		EP	W.Price.No.	Price Range [PLN]		EP
	P min	P max			P min	P max			P min	P max	
RST.Price.32	3972.60	3983.05	10.00%	SOM.Price.25	3970.59	4035.71	13.00%	W.Price.3	3397.73	4069.77	7.69%
RST.Price.67	4673.91	4681.82	10.00%	SOM.Price.61	5200.00	5217.39	13.00%	W.Price.5	4510.64	4945.46	7.69%
RST.Price.96	5217.39	5217.39	10.00%	SOM.Price.63	5243.24	5612.25	50.00%	W.Price.6	4967.74	5296.30	15.38%
RST.Price.105	5384.62	5391.30	20.00%	SOM.Price.72	9861.11	12,714.29	25.00%	W.Price.7	5333.33	5725.81	38.46%
RST.Price.116	5571.43	5595.24	20.00%					W.Price.9	6046.51	6545.46	7.69%
RST.Price.144	6250.00	6268.66	10.00%					W.Price.10	6560.00	7187.50	7.69%
RST.Price.195	11,111.11	11,666.67	20.00%					W.Price.14	10,370.37	11,666.67	15.38%
								W.Price.17	17,948.72	17,948.72	7.69%

**Table 7:** Value range for a given homogenous attribute grouping, illustrated using an example homogenous area. Source: own elaboration.

RST.Attr.23.		SOM.Attr.42.		W.Attr.23.	
V_min_ [PLN]	V_max_ [PLN]	V_min_ [PLN]	V_max_ [PLN]	V_min_ [PLN]	V_max_ [PLN]
6424.82	6545.73	6233.22	7141.33	7369.51	7942.21

**Table 8:** Range of distances between the transaction price for the benchmark property no. 32 and the value range, illustrated using an example homogenous area. Source: own elaboration.

The Test Property					
Location on the Floor	Construction Year	Usable Area of Real Estate	No. of Rooms	Building Materials	Transaction Price (P_t_) [PLN]
2	3	3	5	2	6101.27
RST.Attr.23.		SOM.Attr.42.		W.Attr.23.	
V_min_ [PLN]	V_max_ [PLN]	V_min_ [PLN]	V_max_ [PLN]	V_min_ [PLN]	V_max_ [PLN]
6424.82	6545.73	6233.23	7141,33	7369.51	7942.21
Distance (D) [PLN]					
323.56		131.95		1268.24	

**Table 9:** Mean distances between the transaction price for the example benchmark property and the value range for the three models, illustrated using an example homogenous area. Source: own elaboration.

RST.Attr.23.		SOM.Attr.42.		W.Attr.23.	
V_min_ [PLN]	V_max_ [PLN]	V_min_ [PLN]	V_max_ [PLN]	V_min_ [PLN]	V_max_ [PLN]
6424.82	6545.73	6233.22	7141.33	7369.51	7942.21
Mean Distance [PLN]					
278.23		199.13		749.56	

**Table 10:** The major practical advantages and disadvantages of selected methods. Source: own elaboration.

Rough Set Theory	
Advantages	Disadvantages
particularly useful for low-transaction markets; tolerant of high property price and attribute variability; tolerant of missing data; supports any functional correlation between attributes and price; does not require preliminary analysis; no limits related to number of transactions and the number of attributes; no limits related to attribute coding; supports similarity thresholds.	results are highly influenced by the manually set local indiscernibility (similarity) factor; possible multiple alternative choice of representative decisional rule; conditional attributes of properties can be repeatedly duplicated by multiple decisional rules; convergent decisional rules can emerge, diagnosing a set of representative properties.
**Kohonen’s Neural Networks**	
Advantages	Disadvantages
streamlined calculation; efficiency; reproducibility of results; the model of a phenomenon can be reduced in size; identification of hidden correlations in high-dimensional data sets; easily interpretable results.	highly determined by initial matrix size; the need to set the target features for network training, e.g., maximum number of iterations, cluster size, the variability of Quantization Error; the assumption that a phenomenon can be reduced to a 2D model can sometimes be naively optimistic; discontinuity of the neighborhood of knots, solved at the edges of the SOMs grid, in the classic Kohonen approach.
**Ward’s Method**	
Advantages	Disadvantages
no need to set a starting number of clusters; results are fairly easy to interpret compared to other methods; supports identification of subgroups within clusters, unlike non-hierarchical methods (which provide a “flat” representation of the set); non-complex determination of cluster numbers; simple visualization.	for large sets of data, the calculations may be time-consuming or even unachievable; cluster analysis is scaling-friendly, meaning that if different variables are at different scales, the largest ones may overwhelm the distances; hierarchical methods comprise many algorithms producing different results, and we are not able to determine which of these solutions performs best; tends towards small-size clusters → balanced dendrograms with many components.

## Data Availability

Data are contained within the article and Supplementary Material.

## Supplementary Materials

The following supporting information can be downloaded at: https://www.mdpi.com/article/10.3390/ijerph192215114/s1, Table S1: RST.Attr. groupings of homogeneous property attributes sets within a selected homogeneous area; Table S2: RST.Price. groupings of homogeneous prices of property transactions within a selected homogeneous area; Table S3: Matching RST.Attr. groupings of homogeneous property attributes with the RST.Price. homogeneous price groupings within a selected homogeneous area; Table S4: SOM.Attr. groupings of homogeneous property attributes set within a selected homogeneous area; Table S5: SOM.Price. groupings of homogeneous prices of property transactions within a selected homogeneous area; Table S6: Matching COM.Attr. groupings of homogeneous property attributes with the SOM.Price. homogeneous price groupings within a selected homogeneous area; Table S7: W.Attr. groupings of homogeneous property attributes sets within a selected homogeneous area; Table S8: W.Price. groupings of homogeneous prices of property transactions within a selected homoge-neous area; Table S9: Matching W.Attr. groupings of homogeneous property attributes with the W.Price. homogeneous price groupings within a selected homogeneous area.
