# Supplementary material for: Representative Residential Property Model—Soft Computing Solution
Source: Int J Environ Res Public Health. 2022 Nov 16;19(22):15114. doi: 10.3390/ijerph192215114 (PMC9690382; doi:10.3390/ijerph192215114)
Supplement: Supplementary file 1 [file ijerph-19-15114-s001.zip › ijerph-1973604-supplementary.pdf]

Table S1. RST.Attr. groupings of homogeneous property attributes sets within a selected homogeneous area. Source: own elaboration

RST.Attr.No*	TRANSACTION NUMBER																								
**RST.Attr.1**	24	30	31	38	47	54	55	85	91	119	136	170	171	184	191	200	201	216	256	333	415	419	421	441	479	519
**RST.Attr.2**	0	26	37	43	45	46	48	49	50	75	90	116	118	133	139	183	206	209	210	417	424	445	482	513		
**RST.Attr.3**	67	72	89	129	144	146	147	159	181	186	190	192	194	198	203	205	207	229	230	538	564	583	610			
**RST.Attr.4**	69	140	202	237	262	263	264	334	336	337	339	437	439	440	486	488	525	536	573	577						
**RST.Attr.5**	138	182	238	241	259	265	266	267	270	271	438	481	485	489	522	523	524	572								
**RST.Attr.6**	14	63	66	76	77	101	431	432	433	467	468	469	470	473	532	535	574	582								
**RST.Attr.7**	57	175	211	350	354	356	364	366	370	383	384	390	392	396	401	516										
**RST.Attr.8**	36	56	83	97	231	423	442	446	483	484	487	490	514	515	534											
**RST.Attr.9**	121	122	179	247	248	249	254	321	355	358	373	374	377	394												
**RST.Attr.10**	117	131	135	137	145	187	193	208	260	361	575	585														
**RST.Attr.11**	95	156	232	274	276	306	430	545	551	560	568	593														
**RST.Attr.12**	151	152	250	251	288	381	385	402	405	407	409	557														
**RST.Attr.13**	60	81	106	465	472	475	476	477	492	510	579	580														
**RST.Attr.14**	3	28	29	32	44	51	130	132	418	420	443	444														
**RST.Attr.15**	42	71	84	88	212	213	280	331	332	491	603															
**RST.Attr.16**	279	281	346	495	496	497	498	500	501	507																
**RST.Attr.17**	73	239	240	338	570	576	604	605	606	608																
**RST.Attr.18**	2	27	39	40	79	92	174	362	422	565																
**RST.Attr.19**	22	23	65	110	452	453	456	458	511	512																
**RST.Attr.20**	287	325	341	342	349	387	398	400	403																	
**RST.Attr.21**	4	19	275	303	311	312	318	428	600																	
**RST.Attr.22**	25	86	93	447	480	601	602	607																		
**RST.Attr.23**	225	226	351	352	363	379	393	408																		
**RST.Attr.24**	87	99	109	160	161	494	509	578																		
**RST.Attr.25**	12	20	53	70	100	454	455	461																		
**RST.Attr.26**	7	9	34	309	319	328	413	414																		
**RST.Attr.27**	176	177	357	368	369	389	558																			
**RST.Attr.28**	10	317	329	429	539	540	595																			
**RST.Attr.29**	78	111	112	242	457	462	533																			
**RST.Attr.30**	8	304	305	307	310	343	345																			

**RST.Attr.31**	58	227	324	380	395	397																				
**RST.Attr.32**	68	257	258	261	435	521																				
**RST.Attr.33**	128	134	188	520	563	587																				
**RST.Attr.34**	104	252	359	367	378	391																				
**RST.Attr.35**	59	165	199	224	353	412																				
**RST.Attr.36**	173	217	530	586	590																					
**RST.Attr.37**	16	18	114	559	594																					
**RST.Attr.38**	52	80	451	466	478																					
**RST.Attr.39**	282	283	448	449	517																					
**RST.Attr.40**	278	289	290	544	598																					
**RST.Attr.41**	15	62	82	105																						
**RST.Attr.42**	141	169	189	196																						
**RST.Attr.43**	166	167	222	436																						
**RST.Attr.44**	223	375	386	388																						
**RST.Attr.45**	149	150	215	246																						
**RST.Attr.46**	284	499	504	505																						
**RST.Attr.47**	314	315	320	344																						
**RST.Attr.48**	13	21	64	74																						
**RST.Attr.49**	313	326	327	550																						
**RST.Attr.50**	219	502	591																							
**RST.Attr.51**	195	234	235																							
**RST.Attr.52**	322	323	518																							
**RST.Attr.53**	300	301	302																							
**RST.Attr.54**	335	569	571																							
**RST.Attr.55**	228	233	273																							
**RST.Attr.56**	143	185	562																							
**RST.Attr.57**	17	555	567																							
**RST.Attr.58**	120	269	297																							
**RST.Attr.59**	124	596	597																							
**RST.Attr.60**	102	108	162																							
**RST.Attr.61**	125	245	508																							
**RST.Attr.62**	126	127	493																							
**RST.Attr.63**	142	197	609																							

**RST.Attr.64**	5	6	153																							
**RST.Attr.65**	11	107	459																							
**RST.Attr.66**	584	528																								
**RST.Attr.67**	450	220																								
**RST.Attr.68**	268	113																								
**RST.Attr.69**	293	292																								
**RST.Attr.70**	566	546																								
**RST.Attr.71**	427	416																								
**RST.Attr.72**	561	41																								
**RST.Attr.73**	299	296																								
**RST.Attr.74**	553	96																								
**RST.Attr.75**	474	464																								
**RST.Attr.76**	411	221																								
**RST.Attr.77**	581	460																								
**RST.Attr.78**	236	33																								
**RST.Attr.79**	243	214																								
**RST.Attr.80**	588	164																								
**RST.Attr.81**	556	537																								
**RST.Attr.82**	330	255																								
**RST.Attr.83**	592	291																								
**RST.Attr.84**	404	347																								
**RST.Attr.85**	543	541																								
**RST.Attr.86**	399	382																								
**RST.Attr.87**	547	163																								
**RST.Attr.88**	548	295																								
**RST.Attr.89**	549	503																								
**RST.Attr.90**	506	360																								
**RST.Attr.91**	471	463																								
**RST.Attr.92**	589																									
**RST.Attr.93**	410																									
**RST.Attr.94**	406																									
**RST.Attr.95**	552																									
**RST.Attr.96**	426																									

**RST.Attr.97**	434																									
**RST.Attr.98**	526																									
**RST.Attr.99**	554																									
**RST.Attr.100**	527																									
**RST.Attr.101**	529																									
**RST.Attr.102**	531																									
**RST.Attr.103**	542																									
**RST.Attr.104**	425																									
**RST.Attr.105**	376																									
**RST.Attr.106**	218																									
**RST.Attr.107**	371																									
**RST.Attr.108**	1																									
**RST.Attr.109**	35																									
**RST.Attr.110**	61																									
**RST.Attr.111**	94																									
**RST.Attr.112**	98																									
**RST.Attr.113**	103																									
**RST.Attr.114**	115																									
**RST.Attr.115**	123																									
**RST.Attr.116**	148																									
**RST.Attr.117**	154																									
**RST.Attr.118**	155																									
**RST.Attr.119**	157																									
**RST.Attr.120**	158																									
**RST.Attr.121**	168																									
**RST.Attr.122**	172																									
**RST.Attr.123**	178																									
**RST.Attr.124**	180																									
**RST.Attr.125**	365																									
**RST.Attr.126**	348																									
**RST.Attr.127**	340																									
**RST.Attr.128**	316																									
**RST.Attr.129**	308																									

**RST.Attr.130**	298																									
**RST.Attr.131**	372																									
**RST.Attr.132**	294																									
**RST.Attr.133**	285																									
**RST.Attr.134**	277																									
**RST.Attr.135**	272																									
**RST.Attr.136**	253																									
**RST.Attr.137**	244																									
**RST.Attr.138**	204																									
**RST.Attr.139**	286																									
**RST.Attr.140**	599																									

* No. of homogeneous group (property attributes set).

Table S2. RST.Price. groupings of homogeneous prices of property transactions within a selected homogeneous area. Source: own elaboration

RST.Price.No*	TRANSACTION NUMBER										TRANSACTION PRICE [PLN]	
											MIN	MAX
**RST.Price.1**	444	113	189								327,87	1685,39
**RST.Price.2**	597	437	596								2500,00	2500,00
**RST.Price.3**	124	274	482								2790,70	2872,34
**RST.Price.4**	230	229									3000,00	3000,00
**RST.Price.5**	273	192									3026,59	3034,48
**RST.Price.6**	522	118									3061,23	3083,33
**RST.Price.7**	254	88	255								3108,11	3137,93
**RST.Price.8**	201	120	200								3174,60	3181,82
**RST.Price.9**	242	236									3253,97	3260,87
**RST.Price.10**	269	22									3397,73	3409,09
**RST.Price.11**	445	75	268								3437,50	3454,55
**RST.Price.12**	265	208	266								3476,19	3478,26
**RST.Price.13**	98	48									3555,56	3561,64
**RST.Price.14**	607	602									3585,71	3585,71
**RST.Price.15**	178	155	428								3620,69	3642,86
**RST.Price.16**	128	182	153	8							3679,25	3720,00
**RST.Price.17**	502	440									3731,96	3738,10
**RST.Price.18**	520	196									3750,00	3750,00
**RST.Price.19**	67	197	313	489	0						3767,12	3777,78
**RST.Price.20**	190	85	184								3782,61	3784,31
**RST.Price.21**	84	55									3793,10	3795,46
**RST.Price.22**	452	453	610								3809,52	3811,59
**RST.Price.23**	513	424									3813,95	3813,95
**RST.Price.24**	272	27	187	272	276						3837,21	3846,15
**RST.Price.25**	46	35									3857,14	3857,14
**RST.Price.26**	138	93									3886,79	3888,89
**RST.Price.27**	7	64	469								3894,74	3904,76
**RST.Price.28**	83	56	237	144							3913,04	3920,64
**RST.Price.29**	594	198									3945,67	3947,37

**RST.Price.30**	129	99	1	439							3950,00	3953,49
**RST.Price.31**	103	271	601								3962,26	3970,59
**RST.Price.32**	240	188	351								3972,60	3983,05
**RST.Price.33**	205	90	260								4000,00	4000,00
**RST.Price.34**	306	262									4021,74	4035,71
**RST.Price.35**	156	319	458								4054,05	4069,77
**RST.Price.36**	442	256									4085,71	4093,02
**RST.Price.37**	159	34	185	49	40	183					4102,56	4117,65
**RST.Price.38**	186	207	267								4126,98	4130,44
**RST.Price.39**	238	65									4142,86	4150,94
**RST.Price.40**	47	10	418	420							4166,67	4166,67
**RST.Price.41**	72	37	438	431							4170,21	4210,53
**RST.Price.42**	263	137									4239,13	4242,42
**RST.Price.43**	110	38	360								4255,32	4258,93
**RST.Price.44**	239	33									4263,16	4263,16
**RST.Price.45**	421	371									4266,67	4268,29
**RST.Price.46**	556	193	519								4283,33	4288,89
**RST.Price.47**	228	275	528								4302,33	4311,11
**RST.Price.48**	76	3									4315,79	4315,79
**RST.Price.49**	92	36									4333,33	4342,11
**RST.Price.50**	161	12	106								4351,85	4357,14
**RST.Price.51**	109	51									4378,38	4385,97
**RST.Price.52**	145	142	494								4402,99	4416,67
**RST.Price.53**	24	4									4418,60	4418,60
**RST.Price.54**	82	14									4428,57	4433,96
**RST.Price.55**	264	179	485								4456,52	4459,46
**RST.Price.56**	172	5	80								4473,68	4488,37
**RST.Price.57**	337	465	473	292	337						4510,64	4534,88
**RST.Price.58**	44	87	167								4552,63	4561,40
**RST.Price.59**	26	143	611								4577,78	4583,33
**RST.Price.60**	451	432	509	553	15						4590,16	4600,00
**RST.Price.61**	535	354									4603,45	4605,26
**RST.Price.62**	16	18	194								4615,39	4616,67

**RST.Price.63**	257	304	310	510							4622,64	4622,64
**RST.Price.64**	74	116	370	23							4637,68	4642,86
**RST.Price.65**	89	19	604								4648,65	4651,16
**RST.Price.66**	102	107	133								4660,00	4666,67
**RST.Price.67**	105	81									4673,91	4681,82
**RST.Price.68**	206	464	524								4693,88	4705,88
**RST.Price.69**	466	325									4710,53	4711,11
**RST.Price.70**	415	111	112								4716,98	4723,40
**RST.Price.71**	515	63									4729,73	4729,73
**RST.Price.72**	270	69	467								4736,84	4740,74
**RST.Price.73**	545	146									4750,00	4754,10
**RST.Price.74**	455	259	430	79	100	470	209				4760,87	4789,47
**RST.Price.75**	506	68	139	232							4796,30	4814,82
**RST.Price.76**	435	117									4833,33	4833,33
**RST.Price.77**	160	50	195								4836,07	4844,44
**RST.Price.78**	525	9									4857,14	4861,11
**RST.Price.79**	279	296	358	241							4873,42	4891,30
**RST.Price.80**	477	433									4907,41	4911,77
**RST.Price.81**	203	32	96	412							4916,67	4921,88
**RST.Price.82**	491	204									4925,37	4925,93
**RST.Price.83**	141	86									4929,23	4931,04
**RST.Price.84**	593	550	609								4934,21	4945,46
**RST.Price.85**	135	165	181								4967,74	4983,33
**RST.Price.86**	441	31	191	343	345	463	521	527	582	608	5000,00	5000,00
**RST.Price.87**	43	54	481	537	71	315	61	314			5023,26	5076,92
**RST.Price.88**	541	45	108								5087,72	5088,89
**RST.Price.89**	136	210	454								5094,43	5098,04
**RST.Price.90**	488	91									5106,38	5111,11
**RST.Price.91**	605	30	222								5116,28	5128,21
**RST.Price.92**	180	134	434								5138,89	5150,00
**RST.Price.93**	162	147									5161,29	5166,67
**RST.Price.94**	261	11	425								5192,31	5200,00
**RST.Price.95**	472	199									5208,96	5212,77

**RST.Price.96**	379	70									5217,39	5217,39
**RST.Price.97**	53	21									5227,27	5232,56
**RST.Price.98**	28	13									5243,24	5243,90
**RST.Price.99**	567	566									5256,41	5256,41
**RST.Price.100**	375	2	471	478	514	401					5263,16	5275,36
**RST.Price.101**	447	446	486								5294,12	5296,30
**RST.Price.102**	39	216	347	362							5333,33	5333,33
**RST.Price.103**	417	374	554								5342,47	5359,38
**RST.Price.104**	94	60									5377,36	5377,55
**RST.Price.105**	29	243	352	408	544						5384,62	5391,30
**RST.Price.106**	20	249	52	57	539	166					5402,30	5416,67
**RST.Price.107**	342	78	233								5425,53	5431,04
**RST.Price.108**	115	101									5454,55	5454,55
**RST.Price.109**	104	202	372								5468,75	5471,70
**RST.Price.110**	368	119									5479,17	5479,45
**RST.Price.111**	568	339	390								5480,77	5491,80
**RST.Price.112**	386	309	565								5510,20	5515,15
**RST.Price.113**	492	366									5519,23	5519,30
**RST.Price.114**	468	77									5526,32	5526,32
**RST.Price.115**	462	400									5546,30	5549,02
**RST.Price.116**	363	414	311	291	326	327	318	393			5571,43	5595,24
**RST.Price.117**	547	132	560								5606,06	5612,25
**RST.Price.118**	299	290									5619,05	5622,64
**RST.Price.119**	561	150									5633,80	5634,92
**RST.Price.120**	476	123	302								5638,30	5643,56
**RST.Price.121**	423	595	353	131							5675,68	5690,83
**RST.Price.122**	95	443	564								5714,29	5725,81
**RST.Price.123**	158	157	340								5750,00	5764,71
**RST.Price.124**	331	25									5769,23	5769,23
**RST.Price.125**	511	231									5780,49	5781,25
**RST.Price.126**	479	378									5785,71	5789,47
**RST.Price.127**	361	498	295	66	154						5800,00	5810,81
**RST.Price.128**	533	461	523								5813,95	5816,33

**RST.Price.129**	384	449	548	563							5833,33	5833,33
**RST.Price.130**	62	6	258	277	289						5846,15	5857,14
**RST.Price.131**	457	168									5875,93	5882,35
**RST.Price.132**	402	217									5888,89	5890,41
**RST.Price.133**	531	516	551	392	606						5895,52	5918,92
**RST.Price.134**	312	278	422	557	381						5952,38	5961,54
**RST.Price.135**	512	373									5975,61	5987,50
**RST.Price.136**	574	359	459								6000,00	6000,00
**RST.Price.137**	456	406									6046,51	6046,94
**RST.Price.138**	387	578	603	388							6058,82	6089,74
**RST.Price.139**	598	17	328	391	562	500					6111,11	6125,00
**RST.Price.140**	413	303									6136,36	6142,86
**RST.Price.141**	335	212	213	389	399						6153,85	6176,47
**RST.Price.142**	426	308	403	496	497	419					6200,00	6209,30
**RST.Price.143**	396	130									6228,07	6230,56
**RST.Price.144**	59	590									6250,00	6268,66
**RST.Price.145**	333	73	307	341	543						6293,10	6315,79
**RST.Price.146**	357	246									6329,11	6333,33
**RST.Price.147**	480	460									6346,15	6346,15
**RST.Price.148**	585	305	364								6363,64	6363,64
**RST.Price.149**	114	383	397								6376,81	6384,62
**RST.Price.150**	416	177	394	285							6410,26	6428,57
**RST.Price.151**	407	175									6431,82	6434,78
**RST.Price.152**	526	507									6442,31	6442,31
**RST.Price.153**	348	355	540	576	475						6447,37	6462,96
**RST.Price.154**	518	122	251								6478,87	6488,89
**RST.Price.155**	58	569	148	41	42						6518,52	6545,46
**RST.Price.156**	429	253	338								6560,00	6578,95
**RST.Price.157**	235	234									6610,17	6610,17
**RST.Price.158**	559	173									6641,03	6642,86
**RST.Price.159**	385	382									6666,67	6666,67
**RST.Price.160**	140	376	395								6685,39	6700,00
**RST.Price.161**	367	297	487	534							6710,53	6714,29

**RST.Price.162**	398	495	570	495	404						6727,27	6739,13
**RST.Price.163**	555	324									6774,19	6785,71
**RST.Price.164**	572	334									6800,00	6808,51
**RST.Price.165**	380	214	377	286	286	336					6830,51	6849,06
**RST.Price.166**	493	349									6862,75	6862,75
**RST.Price.167**	97	280	530	97	588						6891,57	6923,08
**RST.Price.168**	248	320	356								6944,44	6949,15
**RST.Price.169**	532	411	490	583							6964,29	6975,31
**RST.Price.170**	546	558	584								7000,00	7000,00
**RST.Price.171**	552	244	436								7010,64	7031,25
**RST.Price.172**	484	483									7078,95	7078,95
**RST.Price.173**	317	298									7083,33	7086,96
**RST.Price.174**	405	316									7111,11	7115,39
**RST.Price.175**	542	322	323								7142,86	7142,86
**RST.Price.176**	350	163									7153,85	7156,25
**RST.Price.177**	171	170	600								7166,67	7187,50
**RST.Price.178**	508	549	592								7279,07	7312,50
**RST.Price.179**	174	293	536	152	410						7348,84	7368,42
**RST.Price.180**	571	245	329	579							7405,71	7424,24
**RST.Price.181**	301	300									7452,38	7452,38
**RST.Price.182**	211	121	344	575							7500,00	7500,00
**RST.Price.183**	599	247	321								7532,47	7567,57
**RST.Price.184**	250	252	499								7653,85	7678,16
**RST.Price.185**	369	149	164	365							7758,62	7812,50
**RST.Price.186**	589	409	501								7904,76	7975,00
**RST.Price.187**	581	474									8026,32	8058,82
**RST.Price.188**	332	294	503	281							8181,82	8255,81
**RST.Price.189**	227	427	505	151	450						8360,00	8560,61
**RST.Price.190**	573	517	577	448	573	580					8680,85	9074,07
**RST.Price.191**	176	215	283								9285,71	9433,96
**RST.Price.192**	538	218									9588,52	9610,39
**RST.Price.193**	504	125									9800,00	9861,11
**RST.Price.194**	284	346	529								10370,37	10760,87

**RST.Price.195**	225	226	282	586	287						11111,11	11666,67
**RST.Price.196**	169	288	591	587	330						12234,04	14634,15
**RST.Price.197**	219	223									17948,72	19754,72
**RST.Price.198**	126	127	219								19754,72	20561,22
**RST.Price.199**	224	221									23333,33	25536,59
**RST.Price.200**	220										30794,12	30794,12

* No. of homogeneous group (property transaction prices).

Table S3. Matching RST.Attr. groupings of homogeneous property attributes with the RST.Price. homogeneous price groupings within a selected homogeneous area. Source: own elaboration

RST. Price.No	RST.Attr.No										TRANSACTION PRICE [PLN]	
											MIN	MAX
**RST.Price.1**	RST.Attr.14.	RST.Attr.68.	RST.Attr.42.								327,87	1685,39
**RST.Price.2**	RST.Attr.59.	RST.Attr.4.	RST.Attr.59.								2500,00	2500,00
**RST.Price.3**	RST.Attr.59.	RST.Attr.11.	RST.Attr.2.								2790,70	2872,34
**RST.Price.4**	RST.Attr.3.	RST.Attr.3.									3000,00	3000,00
**RST.Price.5**	RST.Attr.55.	RST.Attr.3.									3026,59	3034,48
**RST.Price.6**	RST.Attr.5.	RST.Attr.2.									3061,23	3083,33
**RST.Price.7**	RST.Attr.9.	RST.Attr.15.	RST.Attr.82.								3108,11	3137,93
**RST.Price.8**	RST.Attr.1.	RST.Attr.58.	RST.Attr.1.								3174,60	3181,82
**RST.Price.9**	RST.Attr.29.	RST.Attr.78.									3253,97	3260,87
**RST.Price.10**	RST.Attr.58.	RST.Attr.19.									3397,73	3409,09
**RST.Price.11**	RST.Attr.2.	RST.Attr.2.	RST.Attr.68.								3437,50	3454,55
**RST.Price.12**	RST.Attr.5.	RST.Attr.10.	RST.Attr.5.								3476,19	3478,26
**RST.Price.13**	RST.Attr.112.	RST.Attr.2.									3555,56	3561,64
**RST.Price.14**	RST.Attr.22.	RST.Attr.22.									3585,71	3585,71
**RST.Price.15**	RST.Attr.123.	RST.Attr.118.	RST.Attr.21.								3620,69	3642,86
**RST.Price.16**	RST.Attr.33.	RST.Attr.5.	RST.Attr.64.	RST.Attr.30.							3679,25	3720,00
**RST.Price.17**	RST.Attr.50.	RST.Attr.4.									3731,96	3738,10
**RST.Price.18**	RST.Attr.33.	RST.Attr.42.									3750,00	3750,00
**RST.Price.19**	RST.Attr.3.	RST.Attr.63.	RST.Attr.49.	RST.Attr.5.	RST.Attr.2.						3767,12	3777,78
**RST.Price.20**	RST.Attr.3.	RST.Attr.1.	RST.Attr.1.								3782,61	3784,31
**RST.Price.21**	RST.Attr.15.	RST.Attr.1.									3793,10	3795,46
**RST.Price.22**	RST.Attr.19.	RST.Attr.19.	RST.Attr.3.								3809,52	3811,59
**RST.Price.23**	RST.Attr.2.	RST.Attr.2.									3813,95	3813,95
**RST.Price.24**	RST.Attr.135.	RST.Attr.18.	RST.Attr.10.	RST.Attr.135.	RST.Attr.11.						3837,21	3846,15
**RST.Price.25**	RST.Attr.2.	RST.Attr.109.									3857,14	3857,14
**RST.Price.26**	RST.Attr.5.	RST.Attr.22.									3886,79	3888,89
**RST.Price.27**	RST.Attr.26.	RST.Attr.48.	RST.Attr.6.								3894,74	3904,76
**RST.Price.28**	RST.Attr.8.	RST.Attr.8.	RST.Attr.4.	RST.Attr.3.							3913,04	3920,64
**RST.Price.29**	RST.Attr.37.	RST.Attr.3.									3945,67	3947,37
**RST.Price.30**	RST.Attr.3.	RST.Attr.24.	RST.Attr.108.	RST.Attr.4.							3950,00	3953,49
**RST.Price.31**	RST.Attr.113.	RST.Attr.5.	RST.Attr.22.								3962,26	3970,59
**RST.Price.32**	RST.Attr.17.	RST.Attr.33.	RST.Attr.23.								3972,60	3983,05

**RST.Price.33**	RST.Attr.3.	RST.Attr.2.	RST.Attr.10.								4000,00	4000,00
**RST.Price.34**	RST.Attr.11.	RST.Attr.4.									4021,74	4035,71
**RST.Price.35**	RST.Attr.11.	RST.Attr.26.	RST.Attr.19.								4054,05	4069,77
**RST.Price.36**	RST.Attr.8.	RST.Attr.1.									4085,71	4093,02
**RST.Price.37**	RST.Attr.3.	RST.Attr.26.	RST.Attr.56.	RST.Attr.2.	RST.Attr.18.	RST.Attr.2.					4102,56	4117,65
**RST.Price.38**	RST.Attr.3.	RST.Attr.3.	RST.Attr.5.								4126,98	4130,44
**RST.Price.39**	RST.Attr.5.	RST.Attr.19.									4142,86	4150,94
**RST.Price.40**	RST.Attr.1.	RST.Attr.28.	RST.Attr.14.	RST.Attr.14.							4166,67	4166,67
**RST.Price.41**	RST.Attr.3.	RST.Attr.2.	RST.Attr.5.	RST.Attr.6.							4170,21	4210,53
**RST.Price.42**	RST.Attr.4.	RST.Attr.10.									4239,13	4242,42
**RST.Price.43**	RST.Attr.19.	RST.Attr.1.	RST.Attr.90.								4255,32	4258,93
**RST.Price.44**	RST.Attr.17.	RST.Attr.78.									4263,16	4263,16
**RST.Price.45**	RST.Attr.1.	RST.Attr.107.									4266,67	4268,29
**RST.Price.46**	RST.Attr.81.	RST.Attr.10.	RST.Attr.1.								4283,33	4288,89
**RST.Price.47**	RST.Attr.55.	RST.Attr.21.	RST.Attr.66.								4302,33	4311,11
**RST.Price.48**	RST.Attr.6.	RST.Attr.14.									4315,79	4315,79
**RST.Price.49**	RST.Attr.18.	RST.Attr.8.									4333,33	4342,11
**RST.Price.50**	RST.Attr.24.	RST.Attr.25.	RST.Attr.13.								4351,85	4357,14
**RST.Price.51**	RST.Attr.24.	RST.Attr.14.									4378,38	4385,97
**RST.Price.52**	RST.Attr.10.	RST.Attr.63.	RST.Attr.24.								4402,99	4416,67
**RST.Price.53**	RST.Attr.1.	RST.Attr.21.									4418,60	4418,60
**RST.Price.54**	RST.Attr.41.	RST.Attr.6.									4428,57	4433,96
**RST.Price.55**	RST.Attr.4.	RST.Attr.9.	RST.Attr.5.								4456,52	4459,46
**RST.Price.56**	RST.Attr.122.	RST.Attr.64.	RST.Attr.38.								4473,68	4488,37
**RST.Price.57**	RST.Attr.4.	RST.Attr.13.	RST.Attr.6.	RST.Attr.69.	RST.Attr.4.						4510,64	4534,88
**RST.Price.58**	RST.Attr.14.	RST.Attr.24.	RST.Attr.43.								4552,63	4561,40
**RST.Price.59**	RST.Attr.2.	RST.Attr.56.									4577,78	4583,33
**RST.Price.60**	RST.Attr.38.	RST.Attr.6.	RST.Attr.24.	RST.Attr.74.	RST.Attr.41.						4590,16	4600,00
**RST.Price.61**	RST.Attr.6.	RST.Attr.7.									4603,45	4605,26
**RST.Price.62**	RST.Attr.37.	RST.Attr.37.	RST.Attr.3.								4615,39	4616,67
**RST.Price.63**	RST.Attr.32.	RST.Attr.30.	RST.Attr.30.	RST.Attr.13.							4622,64	4622,64
**RST.Price.64**	RST.Attr.48.	RST.Attr.2.	RST.Attr.7.	RST.Attr.19.							4637,68	4642,86
**RST.Price.65**	RST.Attr.3.	RST.Attr.21.	RST.Attr.17.								4648,65	4651,16
**RST.Price.66**	RST.Attr.60.	RST.Attr.65.	RST.Attr.2.								4660,00	4666,67
**RST.Price.67**	RST.Attr.41.	RST.Attr.13.	RST.Attr.23.								4673,91	4681,82

**RST.Price.68**	RST.Attr.2.	RST.Attr.75.	RST.Attr.5.								4693,88	4705,88
**RST.Price.69**	RST.Attr.38.	RST.Attr.20.									4710,53	4711,11
**RST.Price.70**	RST.Attr.1.	RST.Attr.29.	RST.Attr.29.								4716,98	4723,40
**RST.Price.71**	RST.Attr.8.	RST.Attr.6.									4729,73	4729,73
**RST.Price.72**	RST.Attr.5.	RST.Attr.4.	RST.Attr.6.								4736,84	4740,74
**RST.Price.73**	RST.Attr.11.	RST.Attr.3.									4750,00	4754,10
**RST.Price.74**	RST.Attr.25.	RST.Attr.5.	RST.Attr.11.	RST.Attr.18.	RST.Attr.25.	RST.Attr.2.	RST.Attr.6.				4760,87	4789,47
**RST.Price.75**	RST.Attr.90.	RST.Attr.32.	RST.Attr.2.	RST.Attr.11.							4796,30	4814,82
**RST.Price.76**	RST.Attr.32.	RST.Attr.10.									4833,33	4833,33
**RST.Price.77**	RST.Attr.24.	RST.Attr.2.	RST.Attr.51.								4836,07	4844,44
**RST.Price.78**	RST.Attr.4.	RST.Attr.26.									4857,14	4861,11
**RST.Price.79**	RST.Attr.16.	RST.Attr.73.	RST.Attr.9.	RST.Attr.5.							4873,42	4891,30
**RST.Price.80**	RST.Attr.13.	RST.Attr.6.									4907,41	4911,77
**RST.Price.81**	RST.Attr.3.	RST.Attr.14.	RST.Attr.74.	RST.Attr.35.							4916,67	4921,88
**RST.Price.82**	RST.Attr.15.	RST.Attr.138.									4925,37	4925,93
**RST.Price.83**	RST.Attr.42.	RST.Attr.22.									4929,23	4931,04
**RST.Price.84**	RST.Attr.11.	RST.Attr.49.	RST.Attr.63.								4934,21	4945,46
**RST.Price.85**	RST.Attr.10.	RST.Attr.35.	RST.Attr.3.								4967,74	4983,33
**RST.Price.86**	RST.Attr.1.	RST.Attr.1.	RST.Attr.1.	RST.Attr.30.	RST.Attr.30.	RST.Attr.91.	RST.Attr.32.	RST.Attr.100.	RST.Attr.66.	RST.Attr.17.	5000,00	5000,00
**RST.Price.87**	RST.Attr.2.	RST.Attr.1.	RST.Attr.5.	RST.Attr.81.	RST.Attr.15.	RST.Attr.47.	RST.Attr.110.	RST.Attr.47.			5023,26	5076,92
**RST.Price.88**	RST.Attr.85.	RST.Attr.2.	RST.Attr.60.								5087,72	5088,89
**RST.Price.89**	RST.Attr.1.	RST.Attr.2.	RST.Attr.25.								5094,43	5098,04
**RST.Price.90**	RST.Attr.4.	RST.Attr.1.									5106,38	5111,11
**RST.Price.91**	RST.Attr.17.	RST.Attr.1.	RST.Attr.43.								5116,28	5128,21
**RST.Price.92**	RST.Attr.124.	RST.Attr.33.	RST.Attr.97.								5138,89	5150,00
**RST.Price.93**	RST.Attr.60.	RST.Attr.3.									5161,29	5166,67
**RST.Price.94**	RST.Attr.32.	RST.Attr.65.	RST.Attr.104.								5192,31	5200,00
**RST.Price.95**	RST.Attr.13.	RST.Attr.35.									5208,96	5212,77
**RST.Price.96**	RST.Attr.23.	RST.Attr.25.									5217,39	5217,39
**RST.Price.97**	RST.Attr.25.	RST.Attr.48.									5227,27	5232,56
**RST.Price.98**	RST.Attr.14.	RST.Attr.48.									5243,24	5243,90
**RST.Price.99**	RST.Attr.57.	RST.Attr.70.									5256,41	5256,41
**RST.Price.100**	RST.Attr.44.	RST.Attr.18.	RST.Attr.94.	RST.Attr.38.	RST.Attr.8.	RST.Attr.7.					5263,16	5275,36
**RST.Price.101**	RST.Attr.22.	RST.Attr.8.	RST.Attr.4.								5294,12	5296,30
**RST.Price.102**	RST.Attr.18.	RST.Attr.1.	RST.Attr.84.	RST.Attr.18.							5333,33	5333,33

**RST.Price.103**	RST.Attr.2.	RST.Attr.9.	RST.Attr.99.								5342,47	5359,38
**RST.Price.104**	RST.Attr.111.	RST.Attr.13.									5377,36	5377,55
**RST.Price.105**	RST.Attr.14.	RST.Attr.79.	RST.Attr.23.	RST.Attr.23.	RST.Attr.40.						5384,62	5391,30
**RST.Price.106**	RST.Attr.25.	RST.Attr.9.	RST.Attr.38.	RST.Attr.7.	RST.Attr.28.	RST.Attr.43.					5402,30	5416,67
**RST.Price.107**	RST.Attr.20.	RST.Attr.29.	RST.Attr.55.								5425,53	5431,04
**RST.Price.108**	RST.Attr.114.	RST.Attr.6.									5454,55	5454,55
**RST.Price.109**	RST.Attr.34.	RST.Attr.4.	RST.Attr.131.								5468,75	5471,70
**RST.Price.110**	RST.Attr.27.	RST.Attr.1.									5479,17	5479,45
**RST.Price.111**	RST.Attr.11.	RST.Attr.4.	RST.Attr.7.								5480,77	5491,80
**RST.Price.112**	RST.Attr.44.	RST.Attr.26.	RST.Attr.18.								5510,20	5515,15
**RST.Price.113**	RST.Attr.13.	RST.Attr.7.									5519,23	5519,30
**RST.Price.114**	RST.Attr.6.	RST.Attr.6.									5526,32	5526,32
**RST.Price.115**	RST.Attr.29.	RST.Attr.20.									5546,30	5549,02
**RST.Price.116**	RST.Attr.23.	RST.Attr.26.	RST.Attr.21.	RST.Attr.83.	RST.Attr.49.	RST.Attr.49.	RST.Attr.21.	RST.Attr.23.			5571,43	5595,24
**RST.Price.117**	RST.Attr.87.	RST.Attr.14.	RST.Attr.11.								5606,06	5612,25
**RST.Price.118**	RST.Attr.73.	RST.Attr.40.									5619,05	5622,64
**RST.Price.119**	RST.Attr.72.	RST.Attr.45.									5633,80	5634,92
**RST.Price.120**	RST.Attr.13.	RST.Attr.115.	RST.Attr.53.								5638,30	5643,56
**RST.Price.121**	RST.Attr.8.	RST.Attr.28.	RST.Attr.35.	RST.Attr.10.							5675,68	5690,83
**RST.Price.122**	RST.Attr.11.	RST.Attr.14.	RST.Attr.3.								5714,29	5725,81
**RST.Price.123**	RST.Attr.120.	RST.Attr.119.	RST.Attr.127.								5750,00	5764,71
**RST.Price.124**	RST.Attr.15.	RST.Attr.22.									5769,23	5769,23
**RST.Price.125**	RST.Attr.19.	RST.Attr.8.									5780,49	5781,25
**RST.Price.126**	RST.Attr.1.	RST.Attr.34.									5785,71	5789,47
**RST.Price.127**	RST.Attr.10.	RST.Attr.16.	RST.Attr.88.	RST.Attr.6.	RST.Attr.117.						5800,00	5810,81
**RST.Price.128**	RST.Attr.29.	RST.Attr.25.	RST.Attr.5.								5813,95	5816,33
**RST.Price.129**	RST.Attr.7.	RST.Attr.39.	RST.Attr.88.	RST.Attr.33.							5833,33	5833,33
**RST.Price.130**	RST.Attr.41.	RST.Attr.64.	RST.Attr.32.	RST.Attr.134.	RST.Attr.40.						5846,15	5857,14
**RST.Price.131**	RST.Attr.29.	RST.Attr.121.									5875,93	5882,35
**RST.Price.132**	RST.Attr.12.	RST.Attr.36.									5888,89	5890,41
**RST.Price.133**	RST.Attr.102.	RST.Attr.7.	RST.Attr.11.	RST.Attr.7.	RST.Attr.17.						5895,52	5918,92
**RST.Price.134**	RST.Attr.21.	RST.Attr.40.	RST.Attr.18.	RST.Attr.12.	RST.Attr.12.						5952,38	5961,54
**RST.Price.135**	RST.Attr.19.	RST.Attr.9.									5975,61	5987,50
**RST.Price.136**	RST.Attr.6.	RST.Attr.364.	RST.Attr.65.								6000,00	6000,00
**RST.Price.137**	RST.Attr.19.	RST.Attr.94.									6046,51	6046,94

**RST.Price.138**	RST.Attr.20.	RST.Attr.24.	RST.Attr.15.	RST.Attr.44.							6058,82	6089,74
**RST.Price.139**	RST.Attr.40.	RST.Attr.57.	RST.Attr.26.	RST.Attr.34.	RST.Attr.56.	RST.Attr.16.					6111,11	6125,00
**RST.Price.140**	RST.Attr.26.	RST.Attr.21.									6136,36	6142,86
**RST.Price.141**	RST.Attr.54.	RST.Attr.15.	RST.Attr.15.	RST.Attr.27.	RST.Attr.86.						6153,85	6176,47
**RST.Price.142**	RST.Attr.96.	RST.Attr.129.	RST.Attr.20.	RST.Attr.16.	RST.Attr.16.	RST.Attr.	RST.Attr.1.				6200,00	6209,30
**RST.Price.143**	RST.Attr.7.	RST.Attr.14.									6228,07	6230,56
**RST.Price.144**	RST.Attr.35.	RST.Attr.23.	RST.Attr.36.								6250,00	6268,66
**RST.Price.145**	RST.Attr.1.	RST.Attr.17.	RST.Attr.30.	RST.Attr.20.	RST.Attr.85.	RST.Attr.					6293,10	6315,79
**RST.Price.146**	RST.Attr.27.	RST.Attr.45.									6329,11	6333,33
**RST.Price.147**	RST.Attr.22.	RST.Attr.77.									6346,15	6346,15
**RST.Price.148**	RST.Attr.10.	RST.Attr.30.	RST.Attr.7.								6363,64	6363,64
**RST.Price.149**	RST.Attr.37.	RST.Attr.7.	RST.Attr.31.								6376,81	6384,62
**RST.Price.150**	RST.Attr.71.	RST.Attr.27.	RST.Attr.9.	RST.Attr.133.							6410,26	6428,57
**RST.Price.151**	RST.Attr.12.	RST.Attr.7.									6431,82	6434,78
**RST.Price.152**	RST.Attr.98.	RST.Attr.16.									6442,31	6442,31
**RST.Price.153**	RST.Attr.126.	RST.Attr.9.	RST.Attr.28.	RST.Attr.17.	RST.Attr.13.						6447,37	6462,96
**RST.Price.154**	RST.Attr.52.	RST.Attr.9.	RST.Attr.12.								6478,87	6488,89
**RST.Price.155**	RST.Attr.31.	RST.Attr.54.	RST.Attr.116.	RST.Attr.72.	RST.Attr.15.						6518,52	6545,46
**RST.Price.156**	RST.Attr.28.	RST.Attr.136.	RST.Attr.17.								6560,00	6578,95
**RST.Price.157**	RST.Attr.51.	RST.Attr.51.									6610,17	6610,17
**RST.Price.158**	RST.Attr.37.	RST.Attr.36.									6641,03	6642,86
**RST.Price.159**	RST.Attr.12.	RST.Attr.86.									6666,67	6666,67
**RST.Price.160**	RST.Attr.4.	RST.Attr.105.	RST.Attr.31.								6685,39	6700,00
**RST.Price.161**	RST.Attr.34.	RST.Attr.58.	RST.Attr.8.	RST.Attr.8.							6710,53	6714,29
**RST.Price.162**	RST.Attr.20.	RST.Attr.16.	RST.Attr.17.	RST.Attr.16.	RST.Attr.84.						6727,27	6739,13
**RST.Price.163**	RST.Attr.57.	RST.Attr.31.									6774,19	6785,71
**RST.Price.164**	RST.Attr.5.	RST.Attr.4.									6800,00	6808,51
**RST.Price.165**	RST.Attr.31.	RST.Attr.79.	RST.Attr.9.	RST.Attr.139.	RST.Attr.139.	RST.Attr.4.					6830,51	6849,06
**RST.Price.166**	RST.Attr.62.	RST.Attr.20.									6862,75	6862,75
**RST.Price.167**	RST.Attr.8.	RST.Attr.15.	RST.Attr.36.	RST.Attr.8.	RST.Attr.80.						6891,57	6923,08
**RST.Price.168**	RST.Attr.9.	RST.Attr.47.	RST.Attr.7.								6944,44	6949,15
**RST.Price.169**	RST.Attr.6.	RST.Attr.76.	RST.Attr.8.	RST.Attr.3.							6964,29	6975,31
**RST.Price.170**	RST.Attr.70.	RST.Attr.27.	RST.Attr.66.								7000,00	7000,00
**RST.Price.171**	RST.Attr.95.	RST.Attr.137.	RST.Attr.43.								7010,64	7031,25
**RST.Price.172**	RST.Attr.8.	RST.Attr.8.									7078,95	7078,95

**RST.Price.173**	RST.Attr.28.	RST.Attr.130.									7083,33	7086,96
**RST.Price.174**	RST.Attr.12.	RST.Attr.128.									7111,11	7115,39
**RST.Price.175**	RST.Attr.103.	RST.Attr.52.	RST.Attr.52.								7142,86	7142,86
**RST.Price.176**	RST.Attr.7.	RST.Attr.87.									7153,85	7156,25
**RST.Price.177**	RST.Attr.1.	RST.Attr.1.	RST.Attr.21.								7166,67	7187,50
**RST.Price.178**	RST.Attr.61.	RST.Attr.89.	RST.Attr.83.								7279,07	7312,50
**RST.Price.179**	RST.Attr.18.	RST.Attr.69.	RST.Attr.4.	RST.Attr.12.	RST.Attr.93.						7348,84	7368,42
**RST.Price.180**	RST.Attr.54.	RST.Attr.61.	RST.Attr.28.	RST.Attr.13.							7405,71	7424,24
**RST.Price.181**	RST.Attr.53.	RST.Attr.53.									7452,38	7452,38
**RST.Price.182**	RST.Attr.7.	RST.Attr.9.	RST.Attr.47.	RST.Attr.10.							7500,00	7500,00
**RST.Price.183**	RST.Attr.140.	RST.Attr.9.	RST.Attr.9.								7532,47	7567,57
**RST.Price.184**	RST.Attr.12.	RST.Attr.34.	RST.Attr.46.								7653,85	7678,16
**RST.Price.185**	RST.Attr.27.	RST.Attr.45.	RST.Attr.80.	RST.Attr.125.							7758,62	7812,50
**RST.Price.186**	RST.Attr.92.	RST.Attr.12.	RST.Attr.16.								7904,76	7975,00
**RST.Price.187**	RST.Attr.77.	RST.Attr.75.									8026,32	8058,82
**RST.Price.188**	RST.Attr.15.	RST.Attr.132.	RST.Attr.89.	RST.Attr.16.							8181,82	8255,81
**RST.Price.189**	RST.Attr.31.	RST.Attr.71.	RST.Attr.46.	RST.Attr.12.	RST.Attr.67.						8360,00	8560,61
**RST.Price.190**	RST.Attr.4.	RST.Attr.39.	RST.Attr.4.	RST.Attr.39.	RST.Attr.4.	RST.Attr.13.					8680,85	9074,07
**RST.Price.191**	RST.Attr.27.	RST.Attr.45.	RST.Attr.39.								9285,71	9433,96
**RST.Price.192**	RST.Attr.3.	RST.Attr.106.									9588,52	9610,39
**RST.Price.193**	RST.Attr.46.	RST.Attr.61.									9800,00	9861,11
**RST.Price.194**	RST.Attr.46.	RST.Attr.16.	RST.Attr.101.								10370,37	10760,87
**RST.Price.195**	RST.Attr.23.	RST.Attr.23.	RST.Attr.39.	RST.Attr.36.	RST.Attr.20.						11111,11	11666,67
**RST.Price.196**	RST.Attr.42.	RST.Attr.12.	RST.Attr.50.	RST.Attr.33.	RST.Attr.82.						12234,04	14634,15
**RST.Price.197**	RST.Attr.50.	RST.Attr.44.	RST.Attr.62.	RST.Attr.62.	RST.Attr.50.						17948,72	20561,22
**RST.Price.198**	RST.Attr.62.	RST.Attr.62.	RST.Attr.50.								19754,72	20561,22
**RST.Price.199**	RST.Attr.35.	RST.Attr.76.									23333,33	25536,59
**RST.Price.200**	RST.Attr.67.										30794,12	30794,12

Table S4. SOM.Attr. groupings of homogeneous property attributes set within a selected homogeneous area. Source: own elaboration

**SOM.Attr.No***	**TRANSACTION NUMBER**																																					
**SOM.Attr.1**	10	17	285	308	317	329	425	429	539	540	546	555	566	567	595	599																						
**SOM.Attr.2**	11	25	52	80	86	93	107	124	277	416	427	434	447	451	459	464	466	474	478	480	596	597	601	602	607													
**SOM.Attr.3**	14	63	66	76	77	101	431	432	433	460	463	467	468	469	470	471	473	532	535	574	581	582																
**SOM.Attr.4**	2	13	21	27	39	40	64	74	79	92	168	174	291	347	362	404	406	422	565	592																		
**SOM.Attr.5**	138	182	238	241	259	265	266	267	270	271	438	481	485	489	522	523	524	572	102	108	162	204	117	131	135	137	145	187	193	208	260	361	575	585				
**SOM.Attr.6**	67	72	89	129	144	146	147	159	181	186	190	192	194	198	203	205	207	229	230	538	564	583	610															
**SOM.Attr.7**	7	9	34	309	319	328	413	414	542																													
**SOM.Attr.8**	73	239	240	294	316	338	426	570	576	604	605	606	608																									
**SOM.Attr.9**	5	6	22	23	65	110	153	452	453	456	458	511	512																									
**SOM.Attr.10**	60	81	106	465	472	475	476	477	492	510	579	580	0	26	37	43	45	46	48	49	50	75	90	116	118	133	139	183	206	209	210	417	424	445	482	513		
**SOM.Attr.11**	166	167	222	436																																		
**SOM.Attr.12**	16	18	42	61	71	84	88	114	163	164	212	213	280	314	315	320	331	332	344	365	491	547	559	588	594	603												
**SOM.Attr.13**	36	56	83	97	231	348	423	442	446	483	484	487	490	514	515	534																						
**SOM.Attr.14**	15	62	68	82	105	257	258	261	372	435	521																											
**SOM.Attr.15**	141	169	189	196	554																																	
**SOM.Attr.16**	120	269	297	589	33	236	272																															
**SOM.Attr.17**	12	20	53	70	78	100	111	112	242	278	289	290	454	455	457	461	462	533	544	598																		
**SOM.Attr.18**	154	158	292	293	296	299																																
**SOM.Attr.19**	104	252	298	335	359	367	378	391	569	571																												
**SOM.Attr.20**	157	195	228	233	234	235	273	541	543	552																												
**SOM.Attr.21**	4	19	96	115	155	275	303	311	312	313	318	326	327	428	550	553	600																					
**SOM.Attr.22**	8	69	140	202	237	262	263	264	300	301	302	304	305	307	310	334	336	337	339	343	345	437	439	440	486	488	525	536	573	577								
**SOM.Attr.23**	103																																					
**SOM.Attr.24**	98	128	134	172	188	371	520	563	587	531																												
**SOM.Attr.25**	255	330	537	556																																		
**SOM.Attr.26**	58	227	284	324	380	395	397	499	504	505																												
**SOM.Attr.27**	24	30	31	38	47	54	55	85	91	95	119	136	156	170	171	184	191	200	201	216	232	256	274	276	306	333	415	419	421	430	441	479	519	545	551	560	568	593
**SOM.Attr.28**	35	41	143	173	185	217	218	530	561	562	586	590																										
**SOM.Attr.29**	223	375	386	388																																		
**SOM.Attr.30**	113	142	197	268	609																																	
**SOM.Attr.31**	360	506																																				

**SOM.Attr.32**	123	94	528	584																																		
**SOM.Attr.33**	526																																					
**SOM.Attr.34**	1	382	399																																			
**SOM.Attr.35**	3	28	29	32	44	51	130	132	322	323	418	420	443	444	518																							
**SOM.Attr.36**	121	122	179	247	248	249	254	321	355	358	373	374	377	394																								
**SOM.Attr.37**	178	376	527																																			
**SOM.Attr.38**	151	152	250	251	288	381	385	402	405	407	409	557	244	295	548	253																						
**SOM.Attr.39**	87	99	109	160	161	494	509	578																														
**SOM.Attr.40**	126	127	493																																			
**SOM.Attr.41**	219	502	591																																			
**SOM.Attr.42**	225	226	351	352	363	379	393	408																														
**SOM.Attr.43**	279	281	346	495	496	497	498	500	501	507																												
**SOM.Attr.44**	214	243																																				
**SOM.Attr.45**	529																																					
**SOM.Attr.46**	287	325	341	342	349	387	398	400	403	282	283	448	449	517																								
**SOM.Attr.47**	503	549																																				
**SOM.Attr.48**	57	175	211	350	354	356	364	366	370	383	384	390	392	396	401	516																						
**SOM.Attr.49**	176	177	357	368	369	389	558																															
**SOM.Attr.50**	148																																					
**SOM.Attr.51**	340																																					
**SOM.Attr.52**	149	150	215	246																																		
**SOM.Attr.53**	180	286	410																																			
**SOM.Attr.54**	59	165	199	224	353	412																																
**SOM.Attr.55**	125	220	221	245	411	450	508																															

* No. of homogeneous group (property attributes set).

Table S5. SOM.Price. groupings of homogeneous prices of property transactions within a selected homogeneous area. Source: own elaboration.

SOM.Price.No*	TRANSACTION NUMBER																																																																													TRANSACTION PRICE [PLN]	
																																																																													MIN	MAX
**SOM.Price.1**	189																																																																												327,87	327,87
**SOM.Price.2**	113	444																																																																										1470,59	1470,59
**SOM.Price.3**	437	596	597																																																																								2500,00	2500,00
**SOM.Price.4**	482																																																																												2790,70	2790,70
**SOM.Price.5**	124	274																																																																										2857,14	2872,34
**SOM.Price.6**	192	229	230																																																																								3000,00	3000,00
**SOM.Price.7**	273																																																																												3034,48	3034,48
**SOM.Price.8**	118	255	522																																																																								3061,23	3108,11
**SOM.Price.9**	88	254	120																																																																								3116,88	3174,60
**SOM.Price.10**	200	201																																																																										3181,82	3181,82
**SOM.Price.11**	236																																																																												3253,97	3253,97
**SOM.Price.12**	242																																																																												3260,87	3260,87
**SOM.Price.13**	22																																																																												3397,73	3397,73
**SOM.Price.14**	269																																																																												3409,09	3409,09
**SOM.Price.15**	75	208	265	266	268	445																																																																							3437,50	3478,26
**SOM.Price.16**	48	98																																																																											3555,56	3561,64
**SOM.Price.17**	602	607																																																																											3585,71	3585,71
**SOM.Price.18**	155	178	428																																																																								3620,69	3642,86
**SOM.Price.19**	182																																																																												3679,25	3679,25
**SOM.Price.20**	128																																																																												3698,63	3698,63
**SOM.Price.21**	8	67	153	196	197	313	440	502	520																																																																			3716,98	3773,59
**SOM.Price.22**	0	27	35	46	55	84	85	184	187	190	272	276	424	452	453	489	513	610																																																											3776,60	3857,14
**SOM.Price.23**	7	56	64	83	93	138	144	237	469																																																																			3886,79	3920,64
**SOM.Price.24**	1	99	103	129	198	271	439	594																																																																			3945,67	3969,70
**SOM.Price.25**	90	188	205	240	260	262	306	351	601																																																																			3970,59	4035,71
**SOM.Price.26**	34	40	49	156	159	183	185	256	319	442	458																																																																	4054,05	4117,65
**SOM.Price.27**	65	186	207	267																																																																									4126,98	4130,44
**SOM.Price.28**	10	37	47	72	238	418	420	438																																																																			4150,94	4190,48
**SOM.Price.29**	137	263	431																																																																								4210,53	4239,13
**SOM.Price.30**	38	110	360																																																																								4255,32	4258,93
**SOM.Price.31**	33	239	371	421																																																																								4263,16	4268,29
**SOM.Price.32**	193	519	528	556																																																																								4283,33	4302,33
**SOM.Price.33**	3	36	76	92	228	275																																																																							4307,69	4342,11
**SOM.Price.34**	12	51	106	109	161																																																																								4351,85	4385,97
**SOM.Price.35**	494																																																																												4402,99	4402,99
**SOM.Price.36**	4	24	142	145																																																																								4416,67	4418,60
**SOM.Price.37**	14	82																																																																											4428,57	4433,96
**SOM.Price.38**	80	172	179	264	485																																																																							4456,52	4476,74
**SOM.Price.39**	5	465																																																																											4488,37	4510,64
**SOM.Price.40**	292	337	473																																																																								4519,23	4526,32
**SOM.Price.41**	44	167																																																																											4552,63	4552,63
**SOM.Price.42**	26	87	143																																																																								4561,40	4583,33
**SOM.Price.43**	15	432	451	509	553																																																																							4590,16	4597,02
**SOM.Price.44**	354	535																																																																											4603,45	4605,26
**SOM.Price.45**	19	23	74	89	116	133	257	304	310	370	510	604	16	18	194																																																														4615,39	4660,00
**SOM.Price.46**	63	69	111	112	146	206	259	270	325	415	455	464	466	467	515	545	81	102	105	107	524																																																							4666,67	4767,44
**SOM.Price.47**	68	79	100	117	139	160	195	209	232	430	435	470	506																																																																4772,73	4838,71
**SOM.Price.48**	50	525																																																																											4844,44	4857,14
**SOM.Price.49**	9	358																																																																											4861,11	4873,42
**SOM.Price.50**	241	279	296																																																																									4878,05	4880,95
**SOM.Price.51**	433	477																																																																											4907,41	4907,41
**SOM.Price.52**	32	96	203	204	412	491																																																																							4916,67	4925,93
**SOM.Price.53**	141																																																																												4929,23	4929,23
**SOM.Price.54**	86	135	165	550	593	609																																																																							4931,04	4969,23
**SOM.Price.55**	31	54	181	191	343	345	441	463	521	527	582	608																																																															4983,33	5023,26
**SOM.Price.56**	43																																																																												5044,44	5044,44
**SOM.Price.57**	61	71	314	315	481	537																																																																							5057,69	5069,44
**SOM.Price.58**	30	45	91	108	136	210	454	488	541	605																																																																			5087,72	5117,65
**SOM.Price.59**	134	147	162	180	222	434																																																																							5128,21	5166,67
**SOM.Price.60**	11	261																																																																											5192,31	5195,65
**SOM.Price.61**	70	199	379	425	472																																																																								5200,00	5217,39
**SOM.Price.62**	21	53																																																																											5227,27	5232,56
**SOM.Price.63**	2	13	20	28	29	39	52	57	60	77	78	94	101	104	115	119	132	166	202	216	233	243	249	291	309	311	318	326	327	339	342	347	352	362	363	366	368	372	374	375	386	390	400	401	414	417	446	447	462	468	471	478	486	492	514	539	544	547	554	560	565	566	567	568	408	393													5243,24	5612,25
**SOM.Price.64**	6	17	25	62	66	95	123	131	150	154	157	158	168	212	213	217	231	258	277	278	289	290	295	299	302	303	312	328	331	335	340	353	359	361	373	378	381	384	387	388	389	391	392	399	402	406	413	422	423	443	449	456	457	459	461	476	479	498	500	511	512	516	523	531	533	548	551	557	561	562	563	564	574	578	595	598	603	606	5619,05	6176,47
**SOM.Price.65**	59	73	114	130	175	177	246	285	305	307	308	333	341	348	355	357	364	383	394	396	397	403	407	416	419	426	460	475	480	496	497	507	526	540	543	576	585	590																																									6200,00	6462,96
**SOM.Price.66**	41	42	58	122	140	148	173	234	235	251	253	338	367	376	382	385	395	429	487	518	534	559	569																																																								6478,87	6710,53
**SOM.Price.67**	97	214	244	248	280	286	297	320	324	334	336	349	356	377	380	398	404	411	490	493	495	530	532	546	552	555	558	570	572	583	584	588																																															6714,29	7015,39
**SOM.Price.68**	163	170	171	298	316	317	322	323	350	405	436	483	484	508	542	592	600																																																														7031,25	7291,67
**SOM.Price.69**	121	152	164	174	211	245	247	250	252	293	300	301	321	329	344	369	410	499	536	549	571	575	579	599																																																							7312,50	7760,00
**SOM.Price.70**	149	151	227	281	294	332	365	409	427	450	474	501	503	505	517	573	577	581	589																																																														7777,78	8723,40
**SOM.Price.71**	176	215	218	283	448	504	538	580																																																																							8888,89	9800,00

**SOM.Price.72**	125	169	225	226	282	284	287	346	529	586	591																																																																				9861,11	12714,29
**SOM.Price.73**	126	127	219	220	221	223	224	288	330	587																																																																						12916,67	30794,12

* No. of homogeneous group (property transaction prices).

Table S6. Matching COM.Attr. groupings of homogeneous property attributes with the SOM.Price. homoge-neous price groupings within a selected homogeneous area. Source: own elaboration.

SOM.Price.No	SOM.Attr.No*																																																																															TRANSACTION PRICE [PLN]	
																																																																														MIN	MAX
**SOM.Price.1**	SOM.Attr.15.																																																																														327,87	327,87
**SOM.Price.2**	SOM.Attr.30.	SOM.Attr.35.																																																																													1470,59	1470,59
**SOM.Price.3**	SOM.Attr.22.	SOM.Attr.2.	SOM.Attr.2.																																																																													2500,00	2500,00
**SOM.Price.4**	SOM.Attr.10.																																																																														2790,70	2790,70
**SOM.Price.5**	SOM.Attr.2.	SOM.Attr.27.																																																																													2857,14	2872,34
**SOM.Price.6**	SOM.Attr.6.	SOM.Attr.6.	SOM.Attr.6.																																																																													3000,00	3000,00
**SOM.Price.7**	SOM.Attr.20.																																																																														3034,48	3034,48
**SOM.Price.8**	SOM.Attr.10.	SOM.Attr.25.	SOM.Attr.5.																																																																												3061,23	3108,11
**SOM.Price.9**	SOM.Attr.12.	SOM.Attr.36.	SOM.Attr.16.																																																																													3116,88	3174,60
**SOM.Price.10**	SOM.Attr.27.	SOM.Attr.27.																																																																													3181,82	3181,82
**SOM.Price.11**	SOM.Attr.16.																																																																														3253,97	3253,97
**SOM.Price.12**	SOM.Attr.17.																																																																														3260,87	3260,87
**SOM.Price.13**	SOM.Attr.9.																																																																														3397,73	3397,73
**SOM.Price.14**	SOM.Attr.16.																																																																														3409,09	3409,09
**SOM.Price.15**	SOM.Attr.10.	SOM.Attr.5.	SOM.Attr.5.	SOM.Attr.5.	SOM.Attr.30.	SOM.Attr.10.																																																																								3437,50	3478,26
**SOM.Price.16**	SOM.Attr.10.	SOM.Attr.24.																																																																													3555,56	3561,64
**SOM.Price.17**	SOM.Attr.2.	SOM.Attr.2.																																																																													3585,71	3585,71
**SOM.Price.18**	SOM.Attr.21.	SOM.Attr.37.	SOM.Attr.21.																																																																													3620,69	3642,86
**SOM.Price.19**	SOM.Attr.5.																																																																														3679,25	3679,25
**SOM.Price.20**	SOM.Attr.24.																																																																														3698,63	3698,63
**SOM.Price.21**	SOM.Attr.22.	SOM.Attr.6.	SOM.Attr.9.	SOM.Attr.15.	SOM.Attr.30.	SOM.Attr.21.	SOM.Attr.22.	SOM.Attr.41.	SOM.Attr.24.																																																																					3716,98	3773,59
**SOM.Price.22**	SOM.Attr.10.	SOM.Attr.4.	SOM.Attr.28.	SOM.Attr.10.	SOM.Attr.27.	SOM.Attr.12.	SOM.Attr.27.	SOM.Attr.27.	SOM.Attr.5.	SOM.Attr.6.	SOM.Attr.16.	SOM.Attr.27.	SOM.Attr.10.	SOM.Attr.9.	SOM.Attr.9.	SOM.Attr.5.	SOM.Attr.10.	SOM.Attr.6.	SOM.Attr.10.																																																							3776,60	3857,14
**SOM.Price.23**	SOM.Attr.7.	SOM.Attr.13.	SOM.Attr.4.	SOM.Attr.13.	SOM.Attr.2.	SOM.Attr.5.	SOM.Attr.6.	SOM.Attr.22.	SOM.Attr.3.																																																																				3886,79	3920,64
**SOM.Price.24**	SOM.Attr.34.	SOM.Attr.39.	SOM.Attr.23.	SOM.Attr.6.	SOM.Attr.6.	SOM.Attr.5.	SOM.Attr.22.	SOM.Attr.12.																																																																				3945,67	3969,70
**SOM.Price.25**	SOM.Attr.10.	SOM.Attr.24.	SOM.Attr.6.	SOM.Attr.8.	SOM.Attr.5.	SOM.Attr.22.	SOM.Attr.27.	SOM.Attr.42.	SOM.Attr.2.																																																																					3970,59	4035,71
**SOM.Price.26**	SOM.Attr.7.	SOM.Attr.4.	SOM.Attr.10.	SOM.Attr.27.	SOM.Attr.6.	SOM.Attr.10.	SOM.Attr.28.	SOM.Attr.27.	SOM.Attr.7.	SOM.Attr.13.	SOM.Attr.9.																																																																			4054,05	4117,65
**SOM.Price.27**	SOM.Attr.9.	SOM.Attr.6.	SOM.Attr.6.	SOM.Attr.5.																																																																											4126,98	4130,44
**SOM.Price.28**	SOM.Attr.1.	SOM.Attr.10.	SOM.Attr.27.	SOM.Attr.6.	SOM.Attr.5.	SOM.Attr.35.	SOM.Attr.35.	SOM.Attr.5.																																																																					4150,94	4190,48
**SOM.Price.29**	SOM.Attr.5.	SOM.Attr.22.	SOM.Attr.3.																																																																													4210,53	4239,13
**SOM.Price.30**	SOM.Attr.27.	SOM.Attr.9.	SOM.Attr.31.																																																																													4255,32	4258,93
**SOM.Price.31**	SOM.Attr.16.	SOM.Attr.8.	SOM.Attr.24.	SOM.Attr.27.																																																																												4263,16	4268,29
**SOM.Price.32**	SOM.Attr.5.	SOM.Attr.27.	SOM.Attr.32.	SOM.Attr.25.																																																																												4283,33	4302,33
**SOM.Price.33**	SOM.Attr.35.	SOM.Attr.13.	SOM.Attr.3.	SOM.Attr.4.	SOM.Attr.20.	SOM.Attr.21.																																																																								4307,69	4342,11
**SOM.Price.34**	SOM.Attr.17.	SOM.Attr.35.	SOM.Attr.10.	SOM.Attr.39.	SOM.Attr.39.																																																																										4351,85	4385,97
**SOM.Price.35**	SOM.Attr.39.																																																																														4402,99	4402,99
**SOM.Price.36**	SOM.Attr.21.	SOM.Attr.27.	SOM.Attr.30.	SOM.Attr.5.																																																																												4416,67	4418,60
**SOM.Price.37**	SOM.Attr.3.	SOM.Attr.14.																																																																													4428,57	4433,96
**SOM.Price.38**	SOM.Attr.2.	SOM.Attr.24.	SOM.Attr.36.	SOM.Attr.22.	SOM.Attr.5.																																																																										4456,52	4476,74
**SOM.Price.39**	SOM.Attr.9.	SOM.Attr.10.																																																																													4488,37	4510,64
**SOM.Price.40**	SOM.Attr.18.	SOM.Attr.22.	SOM.Attr.3.																																																																													4519,23	4526,32
**SOM.Price.41**	SOM.Attr.35.	SOM.Attr.11.																																																																													4552,63	4552,63
**SOM.Price.42**	SOM.Attr.10.	SOM.Attr.39.	SOM.Attr.28.																																																																													4561,40	4583,33
**SOM.Price.43**	SOM.Attr.14.	SOM.Attr.3.	SOM.Attr.2.	SOM.Attr.39.	SOM.Attr.21.																																																																										4590,16	4597,02
**SOM.Price.44**	SOM.Attr.48.	SOM.Attr.3.																																																																													4603,45	4605,26
**SOM.Price.45**	SOM.Attr.21.	SOM.Attr.9.	SOM.Attr.4.	SOM.Attr.6.	SOM.Attr.10.	SOM.Attr.10.	SOM.Attr.14.	SOM.Attr.22.	SOM.Attr.22.	SOM.Attr.48.	SOM.Attr.10.	SOM.Attr.8.	SOM.Attr.12.	SOM.Attr.12.	SOM.Attr.6.																																																													4615,39	4660,00
**SOM.Price.46**	SOM.Attr.3.	SOM.Attr.22.	SOM.Attr.17.	SOM.Attr.17.	SOM.Attr.6.	SOM.Attr.10.	SOM.Attr.5.	SOM.Attr.5.	SOM.Attr.46.	SOM.Attr.27.	SOM.Attr.17.	SOM.Attr.2.	SOM.Attr.2.	SOM.Attr.3.	SOM.Attr.13.	SOM.Attr.27.	SOM.Attr.10.	SOM.Attr.5.	SOM.Attr.14.	SOM.Attr.2.	SOM.Attr.5.																																																						4666,67	4767,44
**SOM.Price.47**	SOM.Attr.14.	SOM.Attr.4.	SOM.Attr.17.	SOM.Attr.5.	SOM.Attr.10.	SOM.Attr.39.	SOM.Attr.20.	SOM.Attr.10.	SOM.Attr.27.	SOM.Attr.27.	SOM.Attr.14.	SOM.Attr.3.	SOM.Attr.31.																																																																	4772,73	4838,71
**SOM.Price.48**	SOM.Attr.10.	SOM.Attr.22.																																																																													4844,44	4857,14
**SOM.Price.49**	SOM.Attr.7.	SOM.Attr.36.																																																																													4861,11	4873,42
**SOM.Price.50**	SOM.Attr.5.	SOM.Attr.43.	SOM.Attr.18.																																																																													4878,05	4880,95
**SOM.Price.51**	SOM.Attr.3.	SOM.Attr.10.																																																																													4907,41	4907,41
**SOM.Price.52**	SOM.Attr.35.	SOM.Attr.21.	SOM.Attr.6.	SOM.Attr.5.	SOM.Attr.54.	SOM.Attr.12.																																																																								4916,67	4925,93
**SOM.Price.53**	SOM.Attr.15.																																																																														4929,23	4929,23
**SOM.Price.54**	SOM.Attr.2.	SOM.Attr.5.	SOM.Attr.54.	SOM.Attr.21.	SOM.Attr.27.	SOM.Attr.30.																																																																								4931,04	4969,23
**SOM.Price.55**	SOM.Attr.27.	SOM.Attr.27.	SOM.Attr.6.	SOM.Attr.27.	SOM.Attr.22.	SOM.Attr.22.	SOM.Attr.27.	SOM.Attr.3.	SOM.Attr.14.	SOM.Attr.37.	SOM.Attr.3.	SOM.Attr.8.																																																																	4983,33	5023,26
**SOM.Price.56**	SOM.Attr.10.																																																																														5044,44	5044,44
**SOM.Price.57**	SOM.Attr.12.	SOM.Attr.12.	SOM.Attr.12.	SOM.Attr.12.	SOM.Attr.5.	SOM.Attr.25.																																																																								5057,69	5069,44
**SOM.Price.58**	SOM.Attr.27.	SOM.Attr.10.	SOM.Attr.27.	SOM.Attr.5.	SOM.Attr.27.	SOM.Attr.10.	SOM.Attr.17.	SOM.Attr.22.	SOM.Attr.20.	SOM.Attr.8.																																																																			5087,72	5117,65
**SOM.Price.59**	SOM.Attr.24.	SOM.Attr.6.	SOM.Attr.5.	SOM.Attr.53.	SOM.Attr.11.	SOM.Attr.2.																																																																							5128,21	5166,67
**SOM.Price.60**	SOM.Attr.2.	SOM.Attr.14.																																																																													5192,31	5195,65
**SOM.Price.61**	SOM.Attr.17.	SOM.Attr.54.	SOM.Attr.42.	SOM.Attr.1.	SOM.Attr.10.																																																																										5200,00	5217,39
**SOM.Price.62**	SOM.Attr.4.	SOM.Attr.17.																																																																													5227,27	5232,56
**SOM.Price.63**	SOM.Attr.4.	SOM.Attr.4.	SOM.Attr.17.	SOM.Attr.35.	SOM.Attr.35.	SOM.Attr.4.	SOM.Attr.2.	SOM.Attr.48.	SOM.Attr.10.	SOM.Attr.3.	SOM.Attr.17.	SOM.Attr.32.	SOM.Attr.3.	SOM.Attr.19.	SOM.Attr.21.	SOM.Attr.27.	SOM.Attr.35.	SOM.Attr.11.	SOM.Attr.22.	SOM.Attr.27.	SOM.Attr.20.	SOM.Attr.44.	SOM.Attr.36.	SOM.Attr.4.	SOM.Attr.7.	SOM.Attr.21.	SOM.Attr.21.	SOM.Attr.21.	SOM.Attr.21.	SOM.Attr.22.	SOM.Attr.46.	SOM.Attr.4.	SOM.Attr.42.	SOM.Attr.4.	SOM.Attr.42.	SOM.Attr.48.	SOM.Attr.49.	SOM.Attr.14.	SOM.Attr.36.	SOM.Attr.29.	SOM.Attr.29.	SOM.Attr.48.	SOM.Attr.46.	SOM.Attr.48.	SOM.Attr.7.	SOM.Attr.10.	SOM.Attr.13.	SOM.Attr.2.	SOM.Attr.17.	SOM.Attr.3.	SOM.Attr.3.	SOM.Attr.2.	SOM.Attr.22.	SOM.Attr.10.	SOM.Attr.13.	SOM.Attr.1.	SOM.Attr.17.	SOM.Attr.12.	SOM.Attr.15.	SOM.Attr.27.	SOM.Attr.4.	SOM.Attr.1.	SOM.Attr.1.	SOM.Attr.27.	SOM.Attr.42.	SOM.Attr.42.													5243,24	5612,25
**SOM.Price.64**	SOM.Attr.9.	SOM.Attr.1.	SOM.Attr.2.	SOM.Attr.14.	SOM.Attr.3.	SOM.Attr.27.	SOM.Attr.32.	SOM.Attr.5.	SOM.Attr.52.	SOM.Attr.18.	SOM.Attr.20.	SOM.Attr.18.	SOM.Attr.4.	SOM.Attr.12.	SOM.Attr.12.	SOM.Attr.28.	SOM.Attr.13.	SOM.Attr.14.	SOM.Attr.2.	SOM.Attr.17.	SOM.Attr.17.	SOM.Attr.17.	SOM.Attr.38.	SOM.Attr.18.	SOM.Attr.22.	SOM.Attr.22.	SOM.Attr.21.	SOM.Attr.7.	SOM.Attr.12.	SOM.Attr.19.	SOM.Attr.51.	SOM.Attr.54.	SOM.Attr.19.	SOM.Attr.5.	SOM.Attr.36.	SOM.Attr.19.	SOM.Attr.38.	SOM.Attr.48.	SOM.Attr.46.	SOM.Attr.29.	SOM.Attr.49.	SOM.Attr.19.	SOM.Attr.48.	SOM.Attr.34.	SOM.Attr.38.	SOM.Attr.4.	SOM.Attr.7.	SOM.Attr.4.	SOM.Attr.13.	SOM.Attr.35.	SOM.Attr.46.	SOM.Attr.9.	SOM.Attr.17.	SOM.Attr.2.	SOM.Attr.17.	SOM.Attr.10.	SOM.Attr.27.	SOM.Attr.43.	SOM.Attr.43.	SOM.Attr.9.	SOM.Attr.9.	SOM.Attr.48.	SOM.Attr.5.	SOM.Attr.24.	SOM.Attr.17.	SOM.Attr.38.	SOM.Attr.27.	SOM.Attr.38.	SOM.Attr.28.	SOM.Attr.28.	SOM.Attr.24.	SOM.Attr.6.	SOM.Attr.3.	SOM.Attr.39.	SOM.Attr.1.	SOM.Attr.17.	SOM.Attr.12.	SOM.Attr.8.	5619,05	6176,47
**SOM.Price.65**	SOM.Attr.54.	SOM.Attr.8.	SOM.Attr.12.	SOM.Attr.35.	SOM.Attr.48.	SOM.Attr.49.	SOM.Attr.52.	SOM.Attr.1.	SOM.Attr.22.	SOM.Attr.22.	SOM.Attr.1.	SOM.Attr.27.	SOM.Attr.46.	SOM.Attr.13.	SOM.Attr.36.	SOM.Attr.49.	SOM.Attr.48.	SOM.Attr.48.	SOM.Attr.36.	SOM.Attr.48.	SOM.Attr.26.	SOM.Attr.46.	SOM.Attr.38.	SOM.Attr.2.	SOM.Attr.27.	SOM.Attr.8.	SOM.Attr.3.	SOM.Attr.10.	SOM.Attr.2.	SOM.Attr.43.	SOM.Attr.43.	SOM.Attr.43.	SOM.Attr.33.	SOM.Attr.1.	SOM.Attr.20.	SOM.Attr.8.	SOM.Attr.5.	SOM.Attr.28.																																									6200,00	6462,96
**SOM.Price.66**	SOM.Attr.28.	SOM.Attr.12.	SOM.Attr.26.	SOM.Attr.36.	SOM.Attr.22.	SOM.Attr.50.	SOM.Attr.28.	SOM.Attr.20.	SOM.Attr.20.	SOM.Attr.38.	SOM.Attr.38.	SOM.Attr.8.	SOM.Attr.19.	SOM.Attr.37.	SOM.Attr.34.	SOM.Attr.38.	SOM.Attr.26.	SOM.Attr.1.	SOM.Attr.13.	SOM.Attr.35.	SOM.Attr.13.	SOM.Attr.12.	SOM.Attr.19.																																																					6478,87	6710,53
**SOM.Price.67**	SOM.Attr.13.	SOM.Attr.44.	SOM.Attr.38.	SOM.Attr.36.	SOM.Attr.12.	SOM.Attr.53.	SOM.Attr.16.	SOM.Attr.12.	SOM.Attr.26.	SOM.Attr.22.	SOM.Attr.22.	SOM.Attr.46.	SOM.Attr.48.	SOM.Attr.36.	SOM.Attr.26.	SOM.Attr.46.	SOM.Attr.4.	SOM.Attr.55.	SOM.Attr.13.	SOM.Attr.40.	SOM.Attr.43.	SOM.Attr.28.	SOM.Attr.3.	SOM.Attr.1.	SOM.Attr.20.	SOM.Attr.1.	SOM.Attr.49.	SOM.Attr.8.	SOM.Attr.5.	SOM.Attr.6.	SOM.Attr.32.	SOM.Attr.12.																																															6714,29	7015,39
**SOM.Price.68**	SOM.Attr.12.	SOM.Attr.27.	SOM.Attr.27.	SOM.Attr.19.	SOM.Attr.8.	SOM.Attr.1.	SOM.Attr.35.	SOM.Attr.35.	SOM.Attr.48.	SOM.Attr.38.	SOM.Attr.11.	SOM.Attr.13.	SOM.Attr.13.	SOM.Attr.55.	SOM.Attr.7.	SOM.Attr.4.	SOM.Attr.21.																																																													7031,25	7291,67
**SOM.Price.69**	SOM.Attr.36.	SOM.Attr.38.	SOM.Attr.12.	SOM.Attr.4.	SOM.Attr.48.	SOM.Attr.55.	SOM.Attr.36.	SOM.Attr.38.	SOM.Attr.19.	SOM.Attr.18.	SOM.Attr.22.	SOM.Attr.22.	SOM.Attr.36.	SOM.Attr.1.	SOM.Attr.12.	SOM.Attr.49.	SOM.Attr.53.	SOM.Attr.26.	SOM.Attr.22.	SOM.Attr.47.	SOM.Attr.19.	SOM.Attr.5.	SOM.Attr.10.	SOM.Attr.1.																																																					7312,50	7760,00
**SOM.Price.70**	SOM.Attr.52.	SOM.Attr.38.	SOM.Attr.26.	SOM.Attr.43.	SOM.Attr.8.	SOM.Attr.12.	SOM.Attr.12.	SOM.Attr.38.	SOM.Attr.2.	SOM.Attr.55.	SOM.Attr.2.	SOM.Attr.43.	SOM.Attr.47.	SOM.Attr.26.	SOM.Attr.46.	SOM.Attr.22.	SOM.Attr.22.	SOM.Attr.3.	SOM.Attr.16.																																																										7777,78	8723,40
**SOM.Price.71**	SOM.Attr.49.	SOM.Attr.52.	SOM.Attr.28.	SOM.Attr.46.	SOM.Attr.46.	SOM.Attr.26.	SOM.Attr.6.	SOM.Attr.10.																																																																						8888,89	9800,00
**SOM.Price.72**	SOM.Attr.55.	SOM.Attr.15.	SOM.Attr.42.	SOM.Attr.42.	SOM.Attr.46.	SOM.Attr.26.	SOM.Attr.46.	SOM.Attr.43.	SOM.Attr.45.	SOM.Attr.28.	SOM.Attr.41.																																																																		9861,11	12714,29
**SOM.Price.73**	SOM.Attr.40.	SOM.Attr.40.	SOM.Attr.41.	SOM.Attr.55.	SOM.Attr.55.	SOM.Attr.29.	SOM.Attr.54.	SOM.Attr.38.	SOM.Attr.25.	SOM.Attr.24.																																																																			12916,67	30794,12

Table S7. W.Attr. groupings of homogeneous property attributes sets within a selected homogeneous area. Source: own elaboration.

W.Attr.No*	TRANSACTION NUMBER																																																
**W.Attr.1**	0	26	36	37	43	45	46	48	49	50	56	75	83	90	97	116	118	133	139	183	206	209	210	231	417	423	424	442	445	446	482	483	484	487	490	513	514	515	534											
**W.Attr.2**	1	10	17	25	86	93	295	298	308	317	329	416	425	427	429	447	480	539	540	548	555	567	595	601	602	607																								
**W.Attr.3**	2	27	39	40	68	79	92	166	167	174	222	257	258	261	347	348	362	365	372	404	406	422	435	436	521	565																								
**W.Attr.4**	3	24	28	29	30	31	32	38	44	47	51	54	55	85	91	119	130	132	136	170	171	184	191	200	201	216	256	333	415	418	419	420	421	441	443	444	479	519												
**W.Attr.5**	4	7	8	9	19	33	34	96	115	236	275	291	303	304	305	307	309	310	311	312	314	315	318	319	320	328	343	344	345	413	414	428	553	592	600														
**W.Attr.6**	5	6	153	154	155	163	272	277	300	301	302	542	547																																				
**W.Attr.7**	11	107	124	278	289	290	434	459	541	543	544	596	597	598	599																																		
**W.Attr.8**	12	20	52	53	70	78	80	100	102	108	111	112	162	214	242	243	244	451	454	455	457	461	462	466	478	526	533																						
**W.Attr.9**	13	15	21	61	62	64	74	82	105	463	471																																						
**W.Attr.10**	14	22	23	63	65	66	76	77	101	110	431	432	433	452	453	456	458	460	464	467	468	469	470	473	474	511	512	532	535	574	581	582																	
**W.Attr.11**	16	18	42	71	73	84	88	114	158	164	168	212	213	239	240	280	292	293	294	296	299	316	331	332	335	338	426	491	546	559	566	569	570	571	576	588	594	603	604	605	606	608							
**W.Attr.12**	35	41	113	143	173	185	204	217	268	527	530	561	562	586	590																																		
**W.Attr.13**	57	175	176	177	180	211	350	354	356	357	364	366	368	369	370	383	384	389	390	392	396	401	516	558																									
**W.Attr.14**	58	104	227	252	284	324	359	367	378	380	382	391	395	397	399	410	499	504	505																														
**W.Attr.15**	59	121	122	148	165	179	199	224	247	248	249	254	321	340	353	355	358	373	374	377	394	412																											
**W.Attr.16**	60	81	87	98	99	106	109	123	160	161	465	472	475	476	477	492	494	509	510	578	579	580																											
**W.Attr.17**	67	69	72	89	103	129	140	142	144	146	147	159	181	186	190	192	194	197	198	202	203	205	207	229	230	237	262	263	264	334	336	337	339	360	437	439	440	486	488	506	525	536	538	564	573	577	583	609	610
**W.Attr.18**	94	120	128	134	141	169	172	188	189	196	255	269	297	330	371	520	528	531	537	554	556	563	584	587	589																								
**W.Attr.19**	95	156	157	228	232	233	273	274	276	285	306	313	326	327	430	545	550	551	552	560	568	593																											
**W.Attr.20**	117	131	135	137	138	145	182	187	193	195	208	218	234	235	238	241	259	260	265	266	267	270	271	361	438	481	485	489	522	523	524	572	575	585															
**W.Attr.21**	125	126	127	178	219	220	221	245	411	450	493	502	508	529	591																																		
**W.Attr.22**	149	150	151	152	215	246	250	251	287	288	322	323	325	341	342	349	381	385	387	398	400	402	403	405	407	409	518	557																					
**W.Attr.23**	223	225	226	351	352	363	375	376	379	386	388	393	408																																				
**W.Attr.24**	253	279	281	282	283	286	346	448	449	495	496	497	498	500	501	503	507	517	549																														

* No. of homogeneous group (property attributes set).

Table S8. W.Price. groupings of homogeneous prices of property transactions within a selected homoge-neous area. Source: own elaboration.

W.Price.No*	TRANSACTION NUMBER																																																																																				TRANSACTION PRICE [PLN]	
																																																																																				MIN	MAX
W.Price.1	113	189	444																																																																																		327,87	1685,39
W.Price.2	88	118	120	124	192	200	201	229	230	236	242	254	255	273	274	437	482	522	596	597																																																																					2500,00	3260,87
W.Price.3	0	1	7	8	22	27	35	46	48	55	56	64	67	75	83	84	85	90	93	98	99	103	128	129	138	144	153	155	156	178	182	184	187	188	190	196	197	198	205	208	237	240	260	262	265	266	268	269	271	272	276	306	313	319	351	424	428	439	440	445	452	453	458	469	489	502	513	520	594	601	602	607	610													3397,73	4069,77
W.Price.4	3	4	5	10	12	14	24	33	34	36	37	38	40	47	49	51	65	72	76	80	82	92	106	109	110	137	142	145	159	161	172	179	183	185	186	193	207	228	238	239	256	263	264	267	275	360	371	418	420	421	431	438	442	485	494	519	528	556																														4085,71	4488,37
W.Price.5	9	15	16	18	19	23	26	32	44	50	63	68	69	74	79	81	86	87	89	96	100	102	105	107	111	112	116	117	133	139	141	143	146	160	167	194	195	203	204	206	209	232	241	257	259	270	279	292	296	304	310	325	337	354	358	370	412	415	430	432	433	435	451	455	464	465	466	467	470	473	477	491	506	509	510	515	524	525	535	545	550	553	593	604	609	4510,64	4945,46
W.Price.6	2	11	13	21	28	30	31	43	45	53	54	61	70	71	91	108	134	135	136	147	162	165	180	181	191	199	210	222	261	314	315	343	345	375	379	401	425	434	441	446	447	454	463	471	472	478	481	486	488	514	521	527	537	541	566	567	582	605	608																												4967,74	5296,30
W.Price.7	20	29	39	52	57	60	77	78	94	95	101	104	115	119	123	131	132	150	166	202	216	233	243	249	290	291	299	302	309	311	318	326	327	339	342	347	352	353	362	363	366	368	372	374	386	390	400	414	417	423	443	462	468	476	492	539	544	547	554	560	561	564	565	568	595	393	408																			5333,33	5725,81
W.Price.8	6	25	62	66	154	157	158	168	217	231	258	277	278	289	295	312	331	340	359	361	373	378	381	384	392	402	422	449	457	459	461	479	498	511	512	516	523	531	533	548	551	557	563	574	606																																									5750,00	6000,00
W.Price.9	17	41	42	58	59	73	114	122	130	148	175	177	212	213	246	251	285	303	305	307	308	328	333	335	341	348	355	357	364	383	387	388	389	391	394	396	397	399	403	406	407	413	416	419	426	456	460	475	480	496	497	500	507	518	526	540	543	562	569	576	578	585	590	598	603																					6046,51	6545,46
W.Price.10	97	140	163	170	171	173	214	234	235	244	248	253	280	286	297	298	316	317	320	322	323	324	334	336	338	349	350	356	367	376	377	380	382	385	395	398	404	405	411	429	436	483	484	487	490	493	495	530	532	534	542	546	552	555	558	559	570	572	583	584	588	600																								6560,00	7187,50
W.Price.11	121	149	152	164	174	211	245	247	250	252	293	300	301	321	329	344	365	369	409	410	474	499	501	508	536	549	571	575	579	581	589	592	599																																																						7279,07	8058,82
W.Price.12	151	227	281	294	332	427	448	450	503	505	517	573	577	580																																																																										8181,82	9074,07
W.Price.13	125	176	215	218	283	504	538																																																																																	9285,71	9861,11
W.Price.14	225	226	282	284	287	346	529	586																																																																															10370,37	11666,67
W.Price.15	169	288	587	591																																																																																					12234,04	13461,54
W.Price.16	330																																																																																								14634,15	14634,15
W.Price.17	223																																																																																								17948,72	17948,72
W.Price.18	126	127	219																																																																																					19754,72	20561,22
W.Price.19	224																																																																																								23333,33	23333,33
W.Price.20	221																																																																																								25536,59	25536,59
W.Price.21	220																																																																																								30794,12	30794,12

* No. of homogeneous group (property transaction prices).

Table S9. Matching W.Attr. groupings of homogeneous property attributes with the W.Price. homogeneous price groupings within a selected homogeneous area. Source: own elaboration.

W.Price.No	W.Attr. No																																																																																						TRANSACTION PRICE [PLN]	
																																																																																		MIN	MAX
W.Price.1	W.Attr.12.	W.Attr.18.																																																																																			327,87	1685,39
W.Price.2	W.Attr.11.	W.Attr.1.	W.Attr.18.	W.Attr.7.	W.Attr.17.	W.Attr.4.	W.Attr.4.	W.Attr.17.	W.Attr.17.	W.Attr.5.	W.Attr.8.	W.Attr.15.	W.Attr.18.	W.Attr.19.	W.Attr.19.	W.Attr.17.	W.Attr.1.	W.Attr.20.	W.Attr.7.	W.Attr.7.																																																												2500,00	3260,87
W.Price.3	W.Attr.1.	W.Attr.2.	W.Attr.5.	W.Attr.5.	W.Attr.10.	W.Attr.3.	W.Attr.12.	W.Attr.1.	W.Attr.1.	W.Attr.4.	W.Attr.1.	W.Attr.9.	W.Attr.17.	W.Attr.1.	W.Attr.1.	W.Attr.11.	W.Attr.4.	W.Attr.1.	W.Attr.2.	W.Attr.16.	W.Attr.16.	W.Attr.17.	W.Attr.18.	W.Attr.17.	W.Attr.20.	W.Attr.17.	W.Attr.6.	W.Attr.6.	W.Attr.19.	W.Attr.21.	W.Attr.20.	W.Attr.4.	W.Attr.20.	W.Attr.18.	W.Attr.17.	W.Attr.18.	W.Attr.17.	W.Attr.17.	W.Attr.17.	W.Attr.20.	W.Attr.17.	W.Attr.11.	W.Attr.20.	W.Attr.17.	W.Attr.20.	W.Attr.20.	W.Attr.12.	W.Attr.18.	W.Attr.20.	W.Attr.6.	W.Attr.19.	W.Attr.19.	W.Attr.19.	W.Attr.5.	W.Attr.23.	W.Attr.1.	W.Attr.5.	W.Attr.17.	W.Attr.17.	W.Attr.1.	W.Attr.10.	W.Attr.10.	W.Attr.10.	W.Attr.10.	W.Attr.20.	W.Attr.21.	W.Attr.1.	W.Attr.18.	W.Attr.11.	W.Attr.2.	W.Attr.2.	W.Attr.2.	W.Attr.17.													3397,73	4069,77
W.Price.4	W.Attr.4.	W.Attr.5.	W.Attr.6.	W.Attr.2.	W.Attr.8.	W.Attr.10.	W.Attr.4.	W.Attr.5.	W.Attr.5.	W.Attr.1.	W.Attr.1.	W.Attr.4.	W.Attr.3.	W.Attr.4.	W.Attr.1.	W.Attr.4.	W.Attr.10.	W.Attr.17.	W.Attr.10.	W.Attr.8.	W.Attr.9.	W.Attr.3.	W.Attr.16.	W.Attr.16.	W.Attr.10.	W.Attr.20.	W.Attr.17.	W.Attr.20.	W.Attr.17.	W.Attr.16.	W.Attr.18.	W.Attr.15.	W.Attr.1.	W.Attr.12.	W.Attr.17.	W.Attr.20.	W.Attr.17.	W.Attr.19.	W.Attr.20.	W.Attr.11.	W.Attr.4.	W.Attr.17.	W.Attr.17.	W.Attr.20.	W.Attr.5.	W.Attr.17.	W.Attr.18.	W.Attr.4.	W.Attr.4.	W.Attr.4.	W.Attr.10.	W.Attr.20.	W.Attr.1.	W.Attr.20.	W.Attr.16.	W.Attr.4.	W.Attr.18.	W.Attr.18.																													4085,71	4488,37
W.Price.5	W.Attr.5.	W.Attr.9.	W.Attr.11.	W.Attr.11.	W.Attr.5.	W.Attr.10.	W.Attr.1.	W.Attr.4.	W.Attr.4.	W.Attr.1.	W.Attr.10.	W.Attr.3.	W.Attr.17.	W.Attr.9.	W.Attr.3.	W.Attr.16.	W.Attr.2.	W.Attr.16.	W.Attr.17.	W.Attr.5.	W.Attr.8.	W.Attr.8.	W.Attr.9.	W.Attr.7.	W.Attr.8.	W.Attr.8.	W.Attr.1.	W.Attr.20.	W.Attr.1.	W.Attr.1.	W.Attr.18.	W.Attr.12.	W.Attr.17.	W.Attr.16.	W.Attr.3.	W.Attr.17.	W.Attr.20.	W.Attr.17.	W.Attr.12.	W.Attr.1.	W.Attr.1.	W.Attr.19.	W.Attr.20.	W.Attr.3.	W.Attr.20.	W.Attr.20.	W.Attr.24.	W.Attr.11.	W.Attr.11.	W.Attr.5.	W.Attr.5.	W.Attr.22.	W.Attr.17.	W.Attr.13.	W.Attr.15.	W.Attr.13.	W.Attr.15.	W.Attr.4.	W.Attr.19.	W.Attr.10.	W.Attr.10.	W.Attr.3.	W.Attr.8.	W.Attr.8.	W.Attr.10.	W.Attr.16.	W.Attr.8.	W.Attr.10.	W.Attr.10.	W.Attr.10.	W.Attr.16.	W.Attr.11.	W.Attr.17.	W.Attr.16.	W.Attr.16.	W.Attr.1.	W.Attr.20.	W.Attr.17.	W.Attr.10.	W.Attr.19.	W.Attr.19.	W.Attr.5.	W.Attr.19.	W.Attr.11.	W.Attr.17.	4510,64	4945,46
W.Price.6	W.Attr.3.	W.Attr.7.	W.Attr.9.	W.Attr.9.	W.Attr.4.	W.Attr.4.	W.Attr.4.	W.Attr.1.	W.Attr.1.	W.Attr.8.	W.Attr.4.	W.Attr.9.	W.Attr.8.	W.Attr.11.	W.Attr.4.	W.Attr.8.	W.Attr.18.	W.Attr.20.	W.Attr.4.	W.Attr.17.	W.Attr.8.	W.Attr.15.	W.Attr.13.	W.Attr.17.	W.Attr.4.	W.Attr.15.	W.Attr.1.	W.Attr.3.	W.Attr.3.	W.Attr.5.	W.Attr.5.	W.Attr.5.	W.Attr.5.	W.Attr.23.	W.Attr.23.	W.Attr.13.	W.Attr.2.	W.Attr.7.	W.Attr.4.	W.Attr.1.	W.Attr.2.	W.Attr.8.	W.Attr.9.	W.Attr.9.	W.Attr.16.	W.Attr.8.	W.Attr.20.	W.Attr.17.	W.Attr.17.	W.Attr.1.	W.Attr.3.	W.Attr.12.	W.Attr.18.	W.Attr.7.	W.Attr.11.	W.Attr.2.	W.Attr.10.	W.Attr.11.	W.Attr.11.																												4967,74	5296,30
W.Price.7	W.Attr.8.	W.Attr.4.	W.Attr.3.	W.Attr.8.	W.Attr.13.	W.Attr.16.	W.Attr.10.	W.Attr.8.	W.Attr.18.	W.Attr.19.	W.Attr.10.	W.Attr.14.	W.Attr.5.	W.Attr.4.	W.Attr.16.	W.Attr.20.	W.Attr.4.	W.Attr.22.	W.Attr.3.	W.Attr.17.	W.Attr.4.	W.Attr.19.	W.Attr.8.	W.Attr.15.	W.Attr.7.	W.Attr.5.	W.Attr.11.	W.Attr.6.	W.Attr.5.	W.Attr.5.	W.Attr.5.	W.Attr.19.	W.Attr.19.	W.Attr.17.	W.Attr.22.	W.Attr.3.	W.Attr.23.	W.Attr.15.	W.Attr.3.	W.Attr.23.	W.Attr.13.	W.Attr.13.	W.Attr.3.	W.Attr.15.	W.Attr.23.	W.Attr.13.	W.Attr.22.	W.Attr.5.	W.Attr.1.	W.Attr.1.	W.Attr.4.	W.Attr.8.	W.Attr.10.	W.Attr.16.	W.Attr.16.	W.Attr.2.	W.Attr.7.	W.Attr.6.	W.Attr.18.	W.Attr.19.	W.Attr.12.	W.Attr.17.	W.Attr.3.	W.Attr.19.	W.Attr.2.	W.Attr.23.	W.Attr.23.																			5333,33	5725,81
W.Price.8	W.Attr.6.	W.Attr.2.	W.Attr.9.	W.Attr.10.	W.Attr.6.	W.Attr.19.	W.Attr.11.	W.Attr.11.	W.Attr.12.	W.Attr.1.	W.Attr.3.	W.Attr.6.	W.Attr.7.	W.Attr.7.	W.Attr.2.	W.Attr.5.	W.Attr.11.	W.Attr.15.	W.Attr.14.	W.Attr.20.	W.Attr.15.	W.Attr.14.	W.Attr.22.	W.Attr.13.	W.Attr.13.	W.Attr.22.	W.Attr.3.	W.Attr.24.	W.Attr.8.	W.Attr.7.	W.Attr.8.	W.Attr.4.	W.Attr.24.	W.Attr.10.	W.Attr.10.	W.Attr.13.	W.Attr.20.	W.Attr.18.	W.Attr.8.	W.Attr.2.	W.Attr.19.	W.Attr.22.	W.Attr.18.	W.Attr.10.	W.Attr.11.																																														5750,00	6000,00
W.Price.9	W.Attr.2.	W.Attr.12.	W.Attr.11.	W.Attr.14.	W.Attr.15.	W.Attr.11.	W.Attr.11.	W.Attr.15.	W.Attr.4.	W.Attr.15.	W.Attr.13.	W.Attr.13.	W.Attr.11.	W.Attr.11.	W.Attr.22.	W.Attr.22.	W.Attr.19.	W.Attr.5.	W.Attr.5.	W.Attr.5.	W.Attr.2.	W.Attr.5.	W.Attr.4.	W.Attr.11.	W.Attr.22.	W.Attr.3.	W.Attr.15.	W.Attr.13.	W.Attr.13.	W.Attr.13.	W.Attr.22.	W.Attr.22.	W.Attr.14.	W.Attr.15.	W.Attr.13.	W.Attr.14.	W.Attr.14.	W.Attr.22.	W.Attr.3.	W.Attr.22.	W.Attr.23.	W.Attr.5.	W.Attr.2.	W.Attr.4.	W.Attr.11.	W.Attr.10.	W.Attr.10.	W.Attr.16.	W.Attr.2.	W.Attr.24.	W.Attr.24.	W.Attr.24.	W.Attr.24.	W.Attr.22.	W.Attr.8.	W.Attr.2.	W.Attr.7.	W.Attr.12.	W.Attr.11.	W.Attr.11.	W.Attr.16.	W.Attr.20.	W.Attr.12.	W.Attr.7.	W.Attr.11.																					6046,51	6545,46
W.Price.10	W.Attr.1.	W.Attr.17.	W.Attr.6.	W.Attr.4.	W.Attr.4.	W.Attr.12.	W.Attr.8.	W.Attr.20.	W.Attr.20.	W.Attr.8.	W.Attr.15.	W.Attr.24.	W.Attr.11.	W.Attr.24.	W.Attr.18.	W.Attr.2.	W.Attr.11.	W.Attr.2.	W.Attr.5.	W.Attr.22.	W.Attr.22.	W.Attr.14.	W.Attr.17.	W.Attr.17.	W.Attr.11.	W.Attr.22.	W.Attr.13.	W.Attr.13.	W.Attr.14.	W.Attr.23.	W.Attr.15.	W.Attr.14.	W.Attr.14.	W.Attr.22.	W.Attr.14.	W.Attr.22.	W.Attr.3.	W.Attr.22.	W.Attr.21.	W.Attr.2.	W.Attr.3.	W.Attr.1.	W.Attr.1.	W.Attr.1.	W.Attr.1.	W.Attr.21.	W.Attr.24.	W.Attr.12.	W.Attr.10.	W.Attr.1.	W.Attr.6.	W.Attr.11.	W.Attr.19.	W.Attr.2.	W.Attr.13.	W.Attr.11.	W.Attr.11.	W.Attr.20.	W.Attr.17.	W.Attr.18.	W.Attr.11.	W.Attr.5.																								6560,00	7187,50
W.Price.11	W.Attr.15.	W.Attr.22.	W.Attr.22.	W.Attr.11.	W.Attr.3.	W.Attr.13.	W.Attr.21.	W.Attr.15.	W.Attr.22.	W.Attr.14.	W.Attr.11.	W.Attr.6.	W.Attr.6.	W.Attr.15.	W.Attr.2.	W.Attr.5.	W.Attr.3.	W.Attr.13.	W.Attr.22.	W.Attr.14.	W.Attr.10.	W.Attr.14.	W.Attr.24.	W.Attr.21.	W.Attr.17.	W.Attr.24.	W.Attr.11.	W.Attr.20.	W.Attr.16.	W.Attr.10.	W.Attr.18.	W.Attr.5.	W.Attr.7.																																																											7279,07	8058,82
W.Price.12	W.Attr.22.	W.Attr.14.	W.Attr.24.	W.Attr.11.	W.Attr.11.	W.Attr.2.	W.Attr.24.	W.Attr.21.	W.Attr.24.	W.Attr.14.	W.Attr.24.	W.Attr.17.	W.Attr.17.	W.Attr.16.																																																																														8181,82	9074,07
W.Price.13	W.Attr.21.	W.Attr.13.	W.Attr.22.	W.Attr.20.	W.Attr.24.	W.Attr.14.	W.Attr.17.																																																																																		9285,71	9861,11
W.Price.14	W.Attr.23.	W.Attr.23.	W.Attr.24.	W.Attr.14.	W.Attr.22.	W.Attr.24.	W.Attr.21.	W.Attr.12.																																																																																10370,37	11666,67
W.Price.15	W.Attr.18.	W.Attr.22.	W.Attr.18.	W.Attr.21.																																																																																					12234,04	13461,54
W.Price.16	W.Attr.18.																																																																																									14634,15	14634,15
W.Price.17	W.Attr.23.																																																																																								17948,72	17948,72
W.Price.18	W.Attr.21.	W.Attr.21.	W.Attr.21.																																																																																					19754,72	20561,22
W.Price.19	W.Attr.15.																																																																																									23333,33	23333,33
W.Price.20	W.Attr.21.																																																																																								25536,59	25536,59
W.Price.21	W.Attr.21.																																																																																								30794,12	30794,12
